# Delivering synaptic protein mRNAs via extracellular vesicles ameliorates cognitive impairment in a mouse model of Alzheimer’s disease

**DOI:** 10.1186/s12916-024-03359-2

**Published:** 2024-03-25

**Authors:** Huimin Cai, Yana Pang, Ziye Ren, Xiaofeng Fu, Longfei Jia

**Affiliations:** 1 Innovation Center for Neurological Disorders and Department of Neurology, Xuanwu Hospital, Capital Medical University, National Clinical Research Center for Geriatric Diseases, 45 Changchun St., Beijing, 100053 China; 2 Center of Alzheimer’s Disease, Beijing Institute of Brain Disorders, Capital Medical University, Beijing, China

**Keywords:** Alzheimer’s disease, Synaptic dysfunction, Growth-associated protein 43, Synaptosome-associated protein 25, Extracellular vesicles, Messenger RNAs

## Abstract

**Background:**

Synaptic dysfunction with reduced synaptic protein levels is a core feature of Alzheimer’s disease (AD). Synaptic proteins play a central role in memory processing, learning, and AD pathogenesis. Evidence suggests that synaptic proteins in plasma neuronal-derived extracellular vesicles (EVs) are reduced in patients with AD. However, it remains unclear whether levels of synaptic proteins in EVs are associated with hippocampal atrophy of AD and whether upregulating the expression of these synaptic proteins has a beneficial effect on AD.

**Methods:**

In this study, we included 57 patients with AD and 56 healthy controls. We evaluated their brain atrophy through magnetic resonance imaging using the medial temporal lobe atrophy score. We measured the levels of four synaptic proteins, including synaptosome-associated protein 25 (SNAP25), growth-associated protein 43 (GAP43), neurogranin, and synaptotagmin 1 in both plasma neuronal-derived EVs and cerebrospinal fluid (CSF). We further examined the association of synaptic protein levels with brain atrophy. We also evaluated the levels of these synaptic proteins in the brains of 5×FAD mice. Then, we loaded rabies virus glycoprotein-engineered EVs with messenger RNAs (mRNAs) encoding GAP43 and SNAP25 and administered these EVs to 5×FAD mice. After treatment, synaptic proteins, dendritic density, and cognitive function were evaluated.

**Results:**

The results showed that GAP43, SNAP25, neurogranin, and synaptotagmin 1 were decreased in neuronal-derived EVs but increased in CSF in patients with AD, and the changes corresponded to the severity of brain atrophy. GAP43 and SNAP25 were decreased in the brains of 5×FAD mice. The engineered EVs efficiently and stably delivered these synaptic proteins to the brain, where synaptic protein levels were markedly upregulated. Upregulation of synaptic protein expression could ameliorate cognitive impairment in AD by promoting dendritic density. This marks the first successful delivery of synaptic protein mRNAs via EVs in AD mice, yielding remarkable therapeutic effects.

**Conclusions:**

Synaptic proteins are closely related to AD processes. Delivery of synaptic protein mRNAs via EVs stands as a promising effective precision treatment strategy for AD, which significantly advances the current understanding of therapeutic approaches for the disease.

**Supplementary Information:**

The online version contains supplementary material available at 10.1186/s12916-024-03359-2.

## Background

Alzheimer’s disease (AD) is the predominant form of dementia and the leading cause of disability in the elderly worldwide [1]. The estimated prevalence is 10–30% in people over 65 years of age, placing a growing burden on society [2, 3]. However, current treatments are mostly symptomatic, and the development of a disease-modifying therapy is awaited [4, 5].

In addition to amyloid-β (Aβ) and tau pathology, synaptic dysfunction is also a main pathological feature of AD [6, 7]. Proper synapse function is pivotal for nerve impulse transmission and memory formation and storage [8]. Patients with AD show substantial synapse loss in the neocortical area and hippocampus, with the latter displaying a maximal reduction of nearly 50%, which contributes to learning and memory deficits distinctive of this disease [9, 10]. Synaptic dysfunction is considered one of the earliest pathological events in AD and may precede neuronal degeneration and death [11]. Mounting evidence suggests that synaptic dysfunction, rather than Aβ plaques, neurofibrillary tangles, or neuron loss, has the strongest correlation with cognitive impairment [11, 12]. Furthermore, synaptic dysfunction in AD follows the progression from an initially reversible phase to an irreversible depletion phase [6]. Altogether, these findings imply that early intervention targeting synaptic dysfunction may halt or reverse the development of AD.

Synaptic dysfunction is closely related to synaptic proteins. Among them, synaptosome-associated protein 25 (SNAP25), growth-associated protein 43 (GAP43), neurogranin, and synaptotagmin 1 are widely investigated in neurodegenerative diseases [13, 14]. SNAP25 is a critical component for synaptic vesicle trafficking [15], which is modulated by synaptotagmin 1, a presynaptic calcium sensor [16]. Meanwhile, GAP43 is indispensable for axonal elongation and synaptogenesis [17, 18], and neurogranin, a postsynaptic protein, is implicated in long-term potentiation [19]. An abnormal decrease in expression of these synaptic proteins has been observed in brain tissues in patients with AD [20, 21]. We previously reported that these synaptic proteins (SNAP25, GAP43, neurogranin, and synaptotagmin 1) in neuronal-derived extracellular vesicles (EVs) were significantly lower in AD than in healthy controls, and the combination of these proteins can predict AD 5 to 7 years before symptom appearance [22], indicating their roles as potential therapeutic targets for AD. However, in the pilot experiments for the current study, only two of these four synaptic proteins (SNAP25, GAP43) were found to decrease in 5×FAD mouse brains. We therefore focused on the therapeutic effects of upregulating SNAP25 and GAP43 on 5×FAD mice in this study.

EVs are nano-sized vesicles that are released by various cell types found ubiquitously in the extracellular environment. They are involved in intercellular communication and dispersion of proteins, nucleic acids, and other bioactive substances [23, 24]. Recent research efforts have explored EVs as drug-delivery tools or gene therapy vectors [25]. EVs have the natural ability to cross biological barriers, such as the blood-brain barrier, and escape immunogenic clearance. Additionally, EVs are versatile carriers, in which various constituents such as messenger RNAs (mRNAs), short interfering RNAs (siRNAs), and microRNAs (miRNAs), can be encapsulated and delivered, possibly allowing for multi-target and multi-mechanism interventions [26]. Moreover, EVs are genetically engineerable via surface protein modification. By appending specific proteins/peptides onto the surface of EVs, cell/tissue-targeted delivery can be achieved, thus increasing therapeutic efficiency and reducing systemic toxicity [27]. EVs coated with rabies virus glycoprotein (RVG) peptides, which specifically bind to nicotinic acetylcholine receptors expressed in the brain, exhibit effective central nervous system (CNS)-targeted delivery [28]. mRNA-based therapeutics are recognized as a viable strategy for precision medicine [29]. EVs have emerged as a promising vehicle for mRNA delivery, effectively circumventing the intrinsic inability and potential immunogenicity associated with mRNA. The prevailing approach for loading mRNA into EVs involves transfecting EV-producing cells with plasmids encoding the therapeutic mRNA. The resultant elevated cytoplasmic mRNA concentration proves adequate for the efficient packaging of mRNA into EVs [30]. Furthermore, studies corroborate that mRNA delivered via EVs can undergo translation within recipient cells, thereby yielding therapeutic efficacy [31]. Altogether, these properties make EV-based mRNA therapy a promising option in precision medicine for neurodegenerative diseases, including AD.

In this study, to further confirm our previous findings that reduced synaptic proteins in EVs are associated with the development of AD, we examined the relationship between concentrations of SNAP25, GAP43, neurogranin, and synaptotagmin 1 and degrees of hippocampal atrophy in patients with AD. In addition, we validated the reduction of synaptic proteins in the brain of 5×FAD mouse model. Then, we loaded RVG-engineered EVs with mRNAs encoding these synaptic proteins and administered them to 5×FAD mice. We aimed to determine whether EV-based mRNA delivery could efficiently and stably upregulate the expression of synaptic proteins in the brain of AD mice and whether synaptic protein upregulation could rescue neurological function and subsequently improve cognition in AD mice.

## Methods

### Participants

A total of 57 patients with AD and 56 age- and sex-matched healthy controls were included. Clinical diagnosis of probable AD was established using the 2011 criteria by the National Institute on Aging and Alzheimer’s Association [32]. Cerebrospinal fluid (CSF) P-tau181/Aβ42 > 0.14 and CSF Aβ42 < 500 pg/ml (Additional file 1: Fig. S1) were used as biomarker criteria to further differentiate AD from controls, which were determined based on previously published data from our research group [33] as well as other relevant studies [34]. The study protocol was reviewed and approved by the Institutional Review Board of Xuanwu Hospital, Capital Medical University. All participants or their legal representatives provided written informed consent before enrollment.

### Magnetic resonance imaging (MRI) procedure and brain atrophy assessment

Brain MRI scans were obtained using a 3.0 T SIGNA premier scanner (GE HealthCare, USA) at Xuanwu Hospital, Capital Medical University. Medial temporal lobe atrophy (MTA), including the entorhinal cortex, hippocampus, and perirhinal cortex, is a distinctive characteristic of AD [35, 36]. The degree of MTA observed on MRI scans is highly indicative of the severity of medial temporal degenerative pathology at autopsy [37–39]. MTA can be visually evaluated through an established MTA scoring system [40]. All MRI scans were examined using T1-weighted images in the coronal plane, aligned parallel to the brainstem axis and passing through the aqueduct of Silvius. Both cerebral hemispheres were assessed based on the following criteria: a score of 0 indicating no atrophy, 1 representing a widening of the choroid fissure, 2 denoting further widening of the temporal horn of the lateral ventricle and a slight reduction in hippocampal height, 3 indicating a moderate decrease in hippocampal volume, and 4 indicating end-stage progression with exacerbation of all aforementioned findings [40]. The MTA scores were independently assessed by two experienced neurologists, with each hemisphere of the brain evaluated separately. To enhance the accuracy of the assessments, only participants who demonstrated consistent evaluations by both neurologists and concordant scores across both hemispheres were included.

### Neuronal-derived EV isolation from human blood and protein measurement

Multiple studies have noted the presence of neuronal-derived EVs in the circulation [33, 41], suggesting that neuronal-derived EVs might cross the blood-brain barrier and serve as potential mediators reflecting brain changes in the peripheral circulation. Therefore, we isolated neuronal-derived EVs from the blood to investigate the synaptic protein change in the brains of human participants. Fasting blood samples were collected and stored in polypropylene tubes containing ethylenediaminetetraacetic acid. Neuronal-derived EVs were isolated from the blood according to our previous protocol [33]. In brief, one-half milliliter of the whole-blood samples were immediately processed using the ExoQuick exosome precipitation solution (System Biosciences, USA) to collect the total EVs. Neuronal-derived EVs were subsequently isolated through co-immunoprecipitation using a mouse antihuman neural cell adhesion molecule antibody, which was labeled with biotin using the EZ-Link sulfo-NHS-biotin system (ThermoFisher Scientific, USA). Confirmation of the successful collection of EVs and enrichment of neuronal origin were conducted according to our established protocol [33]. The levels of SNAP25, GAP43, neurogranin, and synaptotagmin 1 in EVs were measured using commercially available enzyme-linked immunosorbent assay (ELISA) kits (SNAP25: Proteintech, Rosemont, IL, USA; GAP43, MyBioSource, San Diego, CA, USA; neurogranin: American Research Products, Waltham, MA, USA and synaptotagmin 1: Abbkine, Wuhan, China).

### CSF collection and protein measurement

After blood collection, CSF samples were immediately obtained via lumbar puncture in accordance with international guidelines [42]. The CSF samples were centrifuged and stored at – 80 °C. The levels of Aβ42, P-tau, and T-tau were determined with the INNOTEST ELISA kit (Fujirebio, Japan). The levels of SNAP25, GAP43, neurogranin, and synaptotagmin 1 in CSF were measured using the same ELISA kits with EVs.

### Cell culture and isolation of EVs from cultured cells

Cell culture and transfection were performed according to our previous protocol [43]. Briefly, the HEK293T cell line was obtained from Beijing Syngentech (China), and cellular identity was confirmed by short tandem repeat profiling. The medium used was Dulbecco’s modified Eagle medium supplemented with 10% (v/v) fetal bovine serum (Gibco, Grand Island, NY, USA). To remove exogenous EVs, the complete medium was ultracentrifuged at 100,000 × g for 18 h according to a published protocol [44]. HEK293T cells were seeded on poly L-lysine-coated 6-well plates (Corning, Corning, NY, USA) at a density of 1 × 10^6^ cells/well and cultured at 37 °C in 5% CO_2_ and 95% air condition. All cells were kept in the same generations (maintained within 10 passages) in each experimental treatment. EVs were isolated from the supernatant of HEK293T cells according to a previously published protocol [45]. Briefly, the supernatant was collected and filtered through a 0.22-mm filter (Merck, Kenilworth, NJ, USA) before undergoing centrifugation at 10,000 × *g* for 30 min. Subsequently, ultracentrifugation was performed at 100,000 × *g* for 1 h to collect the EVs. EVs were then resuspended in phosphate-buffered saline (PBS) and stored at − 80°C for no more than 30 days. All EVs were freeze-thawing for one time in this study. A MycoFluor™ kit (ThermoFisher Scientific, Waltham, MA, USA) was used to confirm that the cells were mycoplasma-free before EV collection. The EVs were confirmed with three positive markers, Alix, tumor susceptibility gene101 (TSG101) as well as CD63, and a negative marker, apolipoprotein B (APOB), using Western blot. The morphology of EVs was examined by negatively stained transmission electron microscopy (JEM-1400plus, JEOL, Japan), and the number and size of EVs were quantified using the NanoSight analysis system (Malvern Panalytical, Malvern, UK) according to published protocols [46, 47].

### Construction of an EV-neural targeting system with GAP43 and SNAP25 overexpression

To deliver *Gap43* and *Snap25* mRNAs to the CNS, we constructed an EV system that can target neural cells according to a published protocol [47]. The sequence encoding RVG peptide (YTIWMPENPRPGTPCDIFTNSRGKRASNG), which specifically binds to the acetylcholine receptor and elicits minimal immunogenicity [47], was transfected into the EVs. Briefly, sequences encoding an HA flag and mouse LAMP-2B (NCBI accession number: NM_001290485.2) with RVG fused to the N-terminus were inserted into a pcDNA3.1 plasmid. On day 3 after passage, 5 μg of vectors with *Rvg* mRNAs were transfected into HEK293T cells using Lipofectamine 3000 (ThermoFisher Scientific) at a concentration of 20 μg/10^6^ cells. Three days after the first transfection, the pcDNA3.1 plasmids encoding mouse GAP43 (NCBI accession number: NM_008083.2) and mouse SNAP25 (NCBI accession number: NM_001291056.1) were transfected into HEK293T cells using the same protocol. Cell culture medium was replaced on day 7 and then culture supernatant was harvested 24 h later. To estimate the effect of transfection on apoptosis of HEK293T cells, Caspase 3 activity was measured on day 8 using a commercial kit (Beyotime, China) following the manufacturer’s instructions. The EVs targeting neural cells overexpressing *Gap43* and *Snap25* (EVs-TNGS) were stored at – 80 °C.

### RNA isolation and real-time quantitative reverse transcription-polymerase chain reaction (RT-qPCR) analysis

The Total Cell/Tissue RNA Isolation Kit (Vazyme, Nanjing, Jiangsu, China) was used to extract total RNA from both cultured cells and brain tissues. RNA yield and purity were evaluated using a Nanodrop 1000 spectrophotometer (Thermo Scientific, Wilmington, DE, USA) (Additional file 1: Fig. S2). Subsequently, cDNA synthesis was performed using 1 μg of RNA with HiScript II Q Select RT SuperMix (Vazyme) for qPCR. The resulting cDNA was analyzed by RT-qPCR using the AceQ Universal SYBR qPCR Master Mix (Vazyme) on a Step-One Plus PCR instrument (Applied Biosystems, Foster, CA, USA). The primers used and method details of RT-qPCR are shown in Additional file 1: Table S1 and Additional file 1: Supplementary Methods, respectively.

### Western blotting

Western blotting was conducted according to a protocol previously described [43]. Briefly, the tissues were lysed with radioimmunoprecipitation assay buffer (Applygen, Beijing, China) for 30 min and then homogenized using an ultrasound treatment at 100 W for 3 min and centrifuged at 12,000 × *g* for 20 min. Protein concentrations were quantified using the bicinchoninic acid assay. The proteins were then denatured with sodium dodecyl sulfate (SDS) protein loading buffer (Applygen) and were separated by SDS-polyacrylamide gel electrophoresis (15 μg per well). Samples were transferred to membranes and immunoblotted with the following primary antibodies: anti-GAPDH antibody (1:2000, Abcam, Cambridge, UK), anti-GAP43 antibody (1:1000, Abcam), anti-LAMP-2 antibody (1:500, Abcam), anti-SNAP25 antibody (1:1000, Abcam), anti-Alix antibody (1:1000, Cell Signaling Technology, Danvers, MA, USA), anti-TSG101 antibody (1:2000, ThermoFisher Scientific), anti-CD63 antibody (1:1000, ThermoFisher Scientific), and anti-APOB antibody (1:1000, ThermoFisher Scientific).

### Measurements of Aβ42 and Aβ40 in mouse brain

The levels of Aβ42 and Aβ40 in the cortex and hippocampus of mice were measured using single-molecule array (Simoa) assay on an HD-X Analyzer (Quanterix, Billerica, MA, USA) with Neurology 3-Plex Assay Kits (Quanterix) according to the instructions by the manufacturer. Furthermore, the results were also confirmed using V-PLEX Aβ Peptide Panel 1 (6E10) Kits (Meso Scale Diagnostics, Rockville, MD, USA).

### EV-TNGS treatment

All experimental procedures were approved by the Animal Care and Use Committee of Xuanwu Hospital, Capital Medical University. The 5×FAD mice were obtained from Jackson Laboratory. The mice were placed in groups of no more than five individuals and kept with a standard 12-h light/dark cycle. As behavioral deficits in 5×FAD mice appear around 4 months, and axonal dystrophy and loss of spines and basal dendrites initiate around 4–6 months in this model [48, 49], we used 6-month-old models for the experiment. Six-month-old male 5×FAD mice and age-matched male C57BL/6 mice (wild-type) were used. To determine the optimal dosage of EV-TNGS treatment, mRNA levels of *Gap43* and *Snap25* were quantified in the brains of 5×FAD mice at various dosages of EV-TNGS treatment prior to subsequent experiments. mRNA levels increase with increasing dosage and reach a near plateau at the dosage of 1 × 10^10^ particles in 0.2 mL of PBS per mouse (Additional file 1: Fig. S3). Therefore, this dosage was selected as the optimal dosage in the following experiments. EVs-TNGS (treatment) or wild-type EVs (control) at this dosage were intravenously administered through the tail vein once every 3 days for 12 days, resulting in a total of four injections. The biodistribution analysis of EVs was conducted according to a published protocol [50]. Briefly, the brain, heart, lungs, liver, kidneys, spleen, and hind leg muscle of mice were harvested after transcardial perfusion with PBS. The organs were then weighed and diced for the RNA collection.

### Histopathology and immunohistochemistry

After perfusion with 4% paraformaldehyde, the brains were removed and fixed with 4% paraformaldehyde overnight. Next, they were dehydrated in an ethanol gradient (50%, 70%, 80%, 90%, 95%, 100%) and xylene. The tissues were embedded in paraffin wax and sliced into 4-μm sections that were mounted on slides. The slides were deparaffinized and rehydrated in xylene and an ethanol gradient. Antigens were retrieved via thermal repair in a citrate buffer. Then, the slides were blocked at room temperature for 30 min and incubated with the primary antibody of GAP43 (1:500, Abcam) or SNAP25 (1:500, Abcam) overnight at 4 °C. After washing, the slides were incubated with secondary anti-rabbit IgG H&L (Cy3) antibody (preabsorbed and used at 1:500, Abcam) for 1 h at room temperature. Finally, the slides were counterstained with DAPI (Beyotime, Shanghai, China) and sealed with anti-fluorescence quenching sealing tablets (Yeasen, Shanghai, China). Images were obtained using a confocal microscope (Leica TCS SP5 II).

### Golgi staining

Golgi staining and dendritic calculations were performed according to a published protocol [51]. To detect the dendritic spines of neurons, Golgi staining was conducted in accordance with the manufacturer’s protocol using the FD Rapid Golgi Stain Kit (FD Neurotechnologies, Inc., Columbia, MD). Briefly, brains were rapidly removed from anesthetized mice and washed with Milli-Q water. Fresh brains were impregnated with a Golgi-Cox solution. After 6 h, the solution was renewed, and the brain tissues were stored in the solution at room temperature in the dark for 2 weeks. The brains were then transferred to a cryoprotectant solution (FD Rapid Golgi Stain Kit) and stored at room temperature in the dark. After 24 h, coronal or sagittal sections (100-μm thick) were cryostat-cut at – 24 °C and mounted on gelatin-coated slides. After air-drying and washing, the slides were stained with a developing solution (FD Rapid Golgi Stain Kit). The stained slides were dehydrated with an ethanol gradient (50%, 75%, 95%, and 100%), cleared with a xylene substitute, and mounted with neutral balsam (Yeasen Biotechnology, Shanghai, China) for microscopic examination. Images were obtained using a Nikon Eclipse Ni-U microscope. For quantification analysis, at least 20 neurons per mouse were randomly selected. To ensure cell integrity (particularly that of dendrites), a Z-series of images of each neuron were taken under high magnification (100×, oil immersion). Dendrites that were tapered to a point were considered intact. Image analysis was performed using the Image J software (National Institutes of Health, Maryland, USA). Seven neurons per mouse were assessed to determine the total length, number of branches of dendrites, and spine density.

### Behavioral experiments

To evaluate the memory function in mice, novel object recognition (NOR) task, novel object location (NOL) task, and Morris water maze (MWM) were conducted 1 month after the last dose of EV treatment according to a published protocol [52]. Mice were habituated to a rectangular open field (40 cm × 40 cm × 35 cm) in the absence of objects 24 h prior to the NOR training. During this 5-min session, mice could freely explore the field while their movements and related parameters (total distance covered [cm], time spent in the center [s], and velocity [cm/s]) were recorded and determined using a TSE Systems device. In the training phase, the mice were positioned in the center of the open field, where two identical objects were placed in opposite corners. Mice could freely interact with the objects for a total of 20 s within a maximum time period of 10 min. The mice were removed from the field, and 1 h later, the mice were reintroduced into the open field with one of the objects being replaced with a novel one. Mice could freely explore the field and interact with the object following the same procedure as that during the training phase. The exploration time was determined as the amount of time the snout was directed at an object within 2 cm or less. The recognition index is expressed as follows: recognition index = *T*_novel_/(*T*_novel_ + *T*_familiar_), where *T*_novel_ and *T*_familiar_ represent the time spent interacting with the novel and familiar objects, respectively. In the NOL task, a similar procedure to the NOR task was conducted, with the exception that two identical objects were used for both the training and testing phases, and one of them was relocated to a novel position in the testing phase. For MWM, the experimental apparatus consisted of a white circular tank (120 cm in diameter) containing white opaque water, which was placed in a test room with unchangeable conditions visible to the testing mice. Within the tank, a transparent platform with a diameter of 10 cm was positioned 1.5 cm beneath the water surface and randomly located in the northeast quadrant. During each trial, the mice were tested in a randomized sequence starting with locations of north, south, east, or west, and were given a maximum time of 60 s to swim. In cases where the mice failed to locate the platform, they were gently guided to it and allowed to keep on it for 15 s. The parameters of latency to find the invisible platform and the percentage of time spent in the correct quadrant were recorded to evaluate the cognitive function of mice.

### Statistics

SPSS Statistics for Windows, version 22.0 (IBM Corp., Armonk, NY, USA) and GraphPad Prism 8 (GraphPad Software, San Diego, CA, USA) were used for statistical analysis. Data were evaluated for normality. If data were not normally distributed, a nonparametric approach was used. Data with multiple groups were analyzed using one-way analysis of variance (ANOVA) with a post hoc Bonferroni test. Two-group comparisons were performed using *t*-test. All measurements and analyses were performed in a blinded manner.

## Results

### Changes of GAP43, neurogranin, SNAP25, and synaptotagmin 1 in EVs and CSF correspond to the severity of hippocampal atrophy

**Fig. 1:**
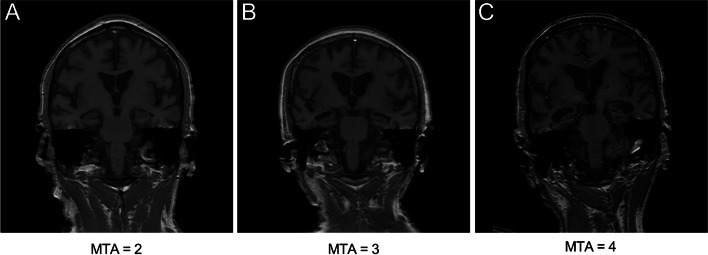
Representative MRI of MTA score rating. Representative T1-weighted images in the coronal plane depicting MTA scores of 2 (**A**), 3 (**B**), and 4 (**C**). MRI, magnetic resonance image; MTA, medial temporal lobe atrophy

**Fig. 2:**
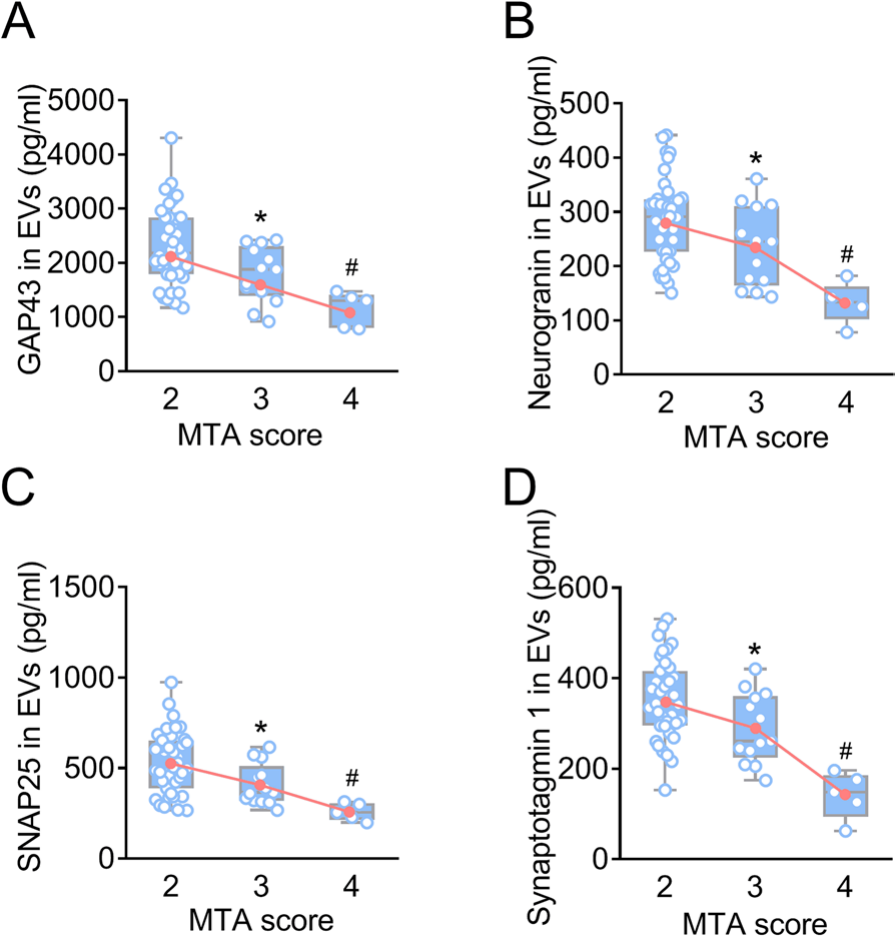
Neuronal-derived EV levels of synaptic proteins in patients with AD. Neuronal-derived EV levels of GAP43 (**A**), neurogranin (**B**), SNAP25 (**C**), and synaptotagmin 1 (**D**) were measured in patients with AD with MTA scores of 2, 3, and 4. A decreasing trend in the mean of synaptic proteins across MTA scores is depicted by the red lines. * Bonferroni-corrected *P* = 2.05 × 10^−2^ (**A**), 4.27 × 10^−2^ (**B**), 2.06 × 10^−2^ (**C**), and 2.27 × 10^−2^ (**D**) compared to MTA = 2 from one way ANOVA test. # Bonferroni-corrected *P* = 9.31 × 10^−4^ (**A**), 7.47 × 10^−5^ (**B**), 1.07 × 10^−3^ (**C**), and 1.22 × 10^−3^ (**D**) compared to MTA = 3 from one way ANOVA test. AD, Alzheimer’s disease; ANOVA, analysis of variance; EV, extracellular vesicle; GAP43, growth-associated protein 43; MTA, medial temporal lobe atrophy; SNAP25, synaptosome-associated protein 25

**Fig. 3:**
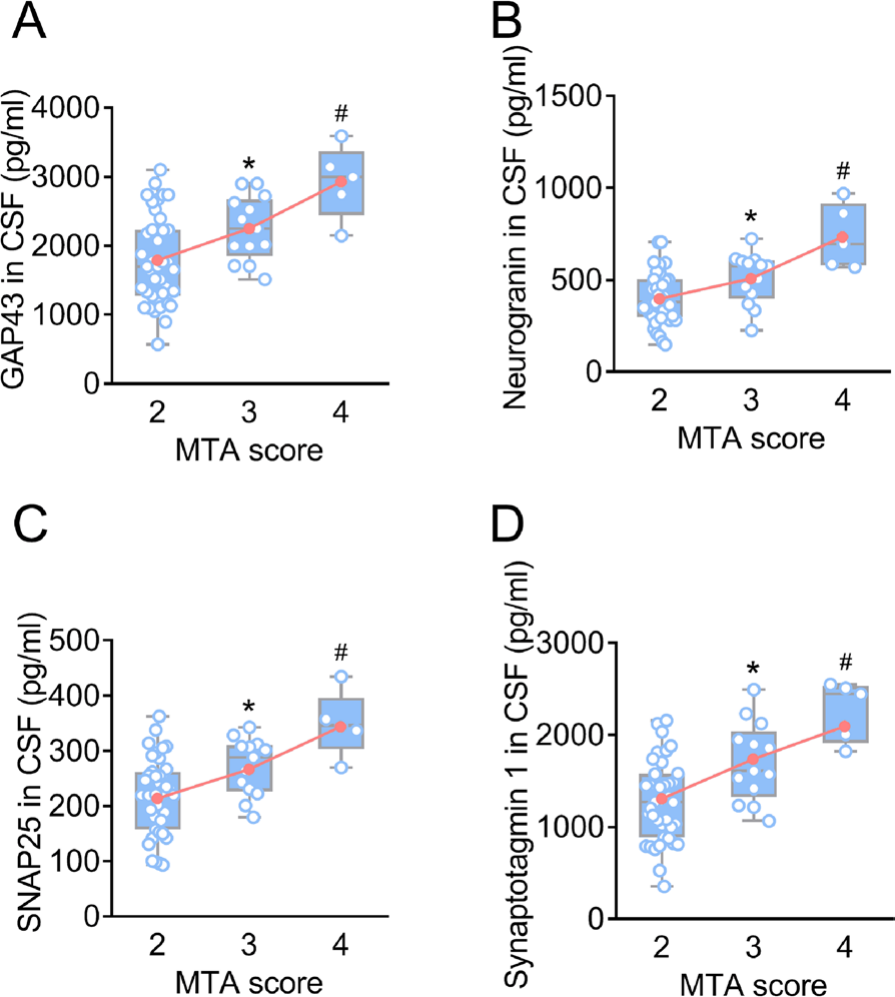
CSF levels of synaptic proteins in patients with AD. CSF levels of GAP43 (**A**), neurogranin (**B**), SNAP25 (**C**), and synaptotagmin 1 (**D**) were measured in patients with AD with MTA scores of 2, 3, and 4. An increasing trend in the mean of synaptic proteins across MTA scores is depicted by the red lines. * Bonferroni-corrected *P* = 2.11 × 10^−2^ (**A**), *P* = 1.79 × 10^−2^ (**B**), 1.56 × 10^−2^ (**C**), and 3.23 × 10^−3^ (**D**) compared to MTA=2 from one way ANOVA test. # Bonferroni-corrected *P* = 4.87 × 10^−4^ (**A**), 1.38 × 10^−5^ (**B**), 2.52 × 10^−4^ (**C**), and 1.65 × 10^−5^ (**D**) compared to MTA = 3 from one way ANOVA test. AD, Alzheimer’s disease; ANOVA, analysis of variance; CSF, cerebrospinal fluid; GAP43, growth-associated protein 43; MTA, medial temporal lobe atrophy; SNAP25, synaptosome-associated protein 25

Table 1 shows the clinical characteristics of patients with AD and controls. The MTA scores of controls were 0 or 1 (Additional file 1: Figs. S4 and S5). These values indicated that there were age-related changes rather than pathological hippocampal atrophy in controls. Figure 1 displays representative images depicting MTA scores of 2, 3, and 4. The MTA scores of AD were 2, 3, and 4 (Figs. 2 and 3), indicating significant hippocampal atrophy. MTA scores show a significant correlation with cognitive performance (Additional file 1: Table S2). To examine whether levels of synaptic proteins are associated with hippocampal atrophy, participants were stratified by MTA scores. In controls, there was no difference in levels of synaptic proteins between MTA scores of 0 and 1 groups (Additional file 1: Figs. S4 and S5), suggesting that under physiological conditions, age-related mild hippocampal atrophy does not lead to changes in synaptic proteins, which may be related to compensatory mechanisms of the body. In AD, there were significant differences in synaptic proteins in both plasma neuronal-derived EVs and CSF between MTA scores of 2, 3, and 4 groups (Figs. 2 and 3). Interestingly, changes in synaptic proteins were correlated with the severity of hippocampal atrophy (for example, the higher MTA scores, the lower synaptic proteins in EVs). These findings suggested that synaptic proteins may be targets for intervention in AD processes.

**Table 1:** Characteristics of participants

Characteristic	Total sample (*n* = 113)	Controls (*n* = 56)	AD (*n* = 57)
Age, mean (SD)	74.6 (6.9)	76.1 (6.4)	73.1 (7.0)
Education year, mean (SD)	9.6 (2.3)	9.9 (2.2)	9.2 (2.4)
Women, No. (%)	57 (50.4)	28 (50.0)	29 (50.9)
*APOE* ε4 positive, No. (%)	36 (31.9)	10 (17.9)	26 (45.6)^a^
MMSE score, mean (SD)	23.0 (6.4)	29.0 (0.6)	17.0 (3.1)^a^
ADAS-Cog, mean (SD)	13.1(9.4)	5.4 (2.4)	20.7 (7.3)^a^
Aβ42 (pg/ml), mean (SD)	539.7 (216.0)	720.4 (146.4)	362.1 (86.4)^a^
P-tau (pg/ml), mean (SD)	91.3 (58.4)	53.1 (23.6)	128.9 (58.1)^a^
T-tau (pg/ml), mean (SD)	485.1 (208.1)	326.7 (79.8)	640.8 (175.0)^a^
MTA score distribution, No. (%)			
MTA = 0	40 (35.4)	40 (71.4)	0 (0)
MTA = 1	16 (14.2)	16 (28.6)	0 (0)
MTA = 2	39 (34.5)	0 (0)	39 (68.4)
MTA = 3	13 (11.5)	0 (0)	13 (22.8)
MTA = 4	5 (4.4)	0 (0)	5 (8.8)

The values of age, education year, MMSE, ADAS-Cog, and cerebrospinal fluid biomarkers are shown as mean (standard deviation).

*Abbreviations*: Aβ amyloid-β, *AD* Alzheimer’s disease, *ADAS-Cog* the Alzheimer’s Disease assessment scale cognitive subscale, *APOE* apolipoprotein E, *MMSE* Mini-Mental State Examination, *MTA* Medial temporal lobe atrophy, *P-tau* Phosphorylated tau, *SD* Standard deviation, *T-tau* Total tau

^a^*P* < 0.05 compared to controls

### GAP43 and SNAP25 levels are reduced in 5×FAD mouse brain

**Fig. 4:**
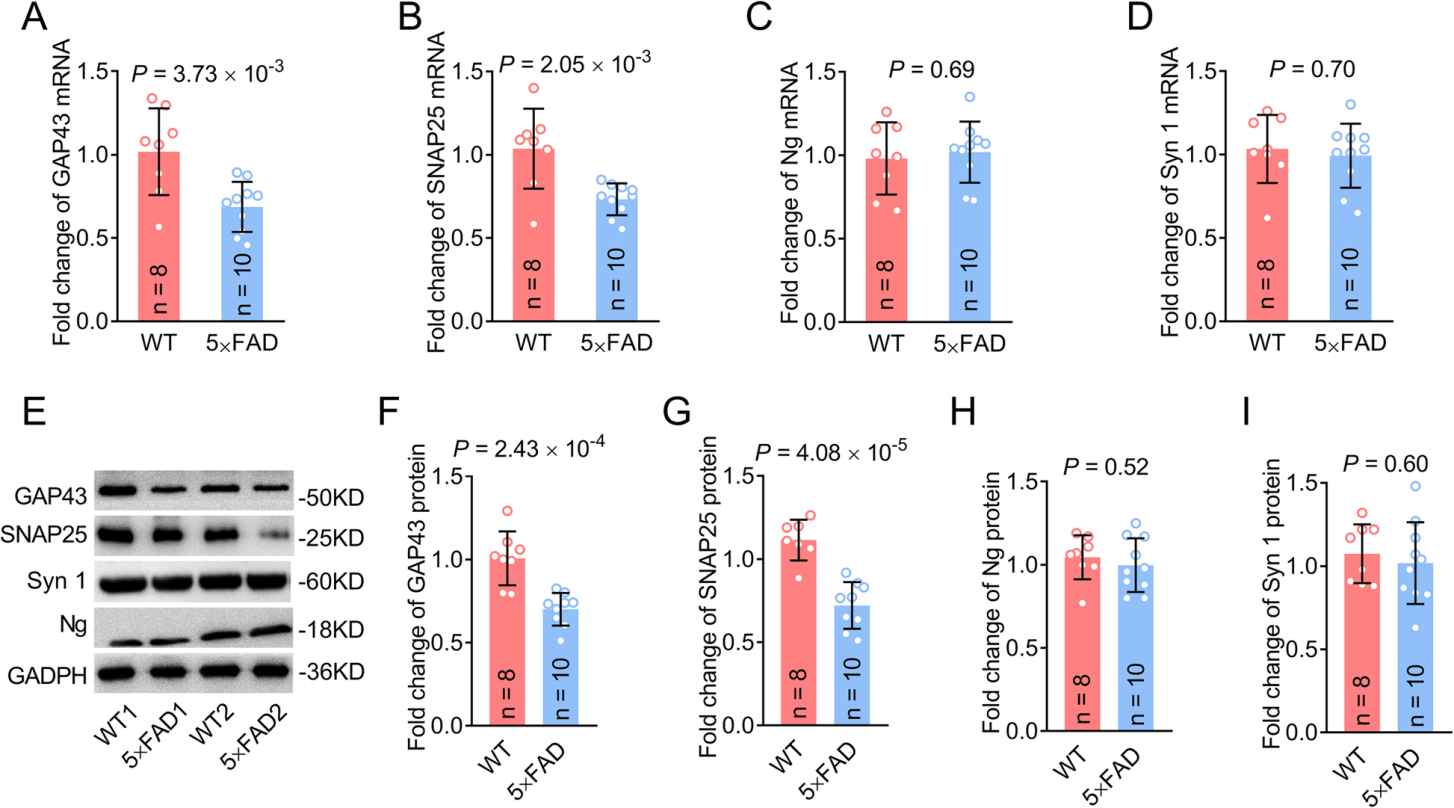
Reduction of GAP43 and SNAP25 levels in the hippocampus of 5×FAD mouse brain. **A-D** *Gap43* (**A**), *Snap25* (**B**), *neurogranin* (**C**), and *synaptotagmin 1*(**D**) mRNA levels quantified using RT-qPCR. **E**–**I** Representative Western blots (**E**) and quantitative assessment (**F**–**I**) of GAP43 (**E** and **F**), SNAP25 (**E** and **G**), neurogranin (**E** and **H**), and synaptotagmin 1 (**E** and **I**) protein levels. Group comparison was performed using t-test. GAP43, growth-associated protein 43; Ng, neurogranin; mRNA, messenger RNA; RT-qPCR, real-time quantitative reverse transcription-polymerase chain reaction; SNAP25, synaptosome-associated protein 25; Syn 1, synaptotagmin 1; WT, wild-type

To further investigate whether synaptic proteins exhibit a similar reduction in the animal model, we detected the levels of GAP43, neurogranin, SNAP25, and synaptotagmin 1 in the brain of 5×FAD mice by western blot and RT-qPCR. As expected, the mRNA and protein levels of GAP43 and SNAP25 were decreased (Fig. 4A, B, and E–G, Additional file 1: Fig. S6). However, contrary to the findings observed in human studies, the levels of neurogranin and synaptotagmin 1 showed no significant difference between 5×FAD and control mice (Fig. 4C–E, H, and I), which may be due to species differences or different stages of the disease between the mice and patients. Altogether, these data suggest that GAP43 and SNAP25 play roles in the pathogenesis of 5×FAD mice.

### Construction of an EV-neural targeting system to overexpress *Gap43* and *Snap25*

**Fig. 5:**
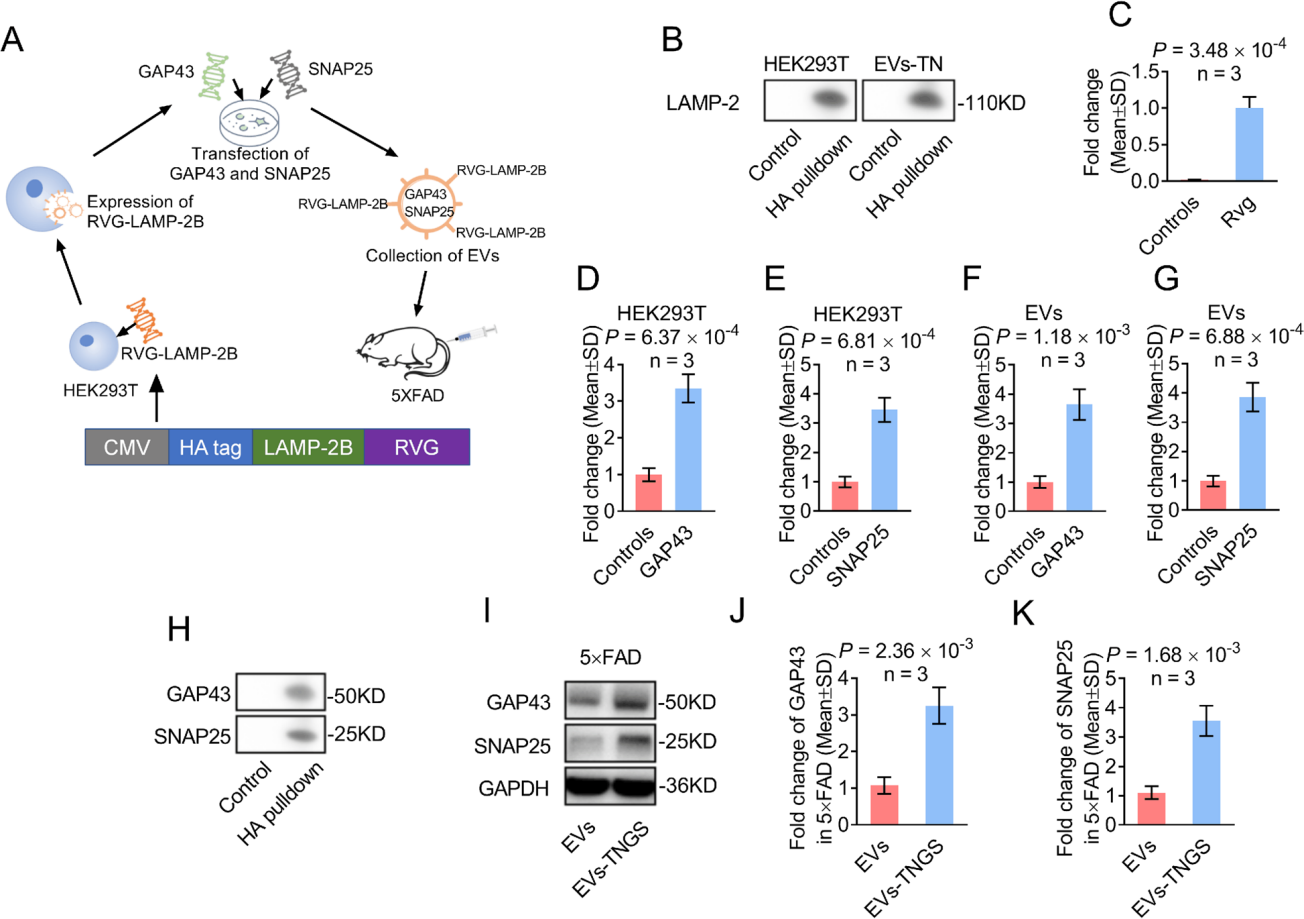
Construction of a neural-cell-targeting EV system loaded with mRNAs of *Gap43* and *Snap25*. **A** Schematic representation of generation, collection, and administration of neural-cell-targeting EVs overexpressing *Gap43* and *Snap25* mRNAs. **B** HA pulldown assays and representative Western blots of LAMP-2B in HEK293T cells transfected with HA-RVG-LAMP-2B plasmid and their corresponding EVs. **C** RT-qPCR of HEK293T cells transfected with HA-RVG-LAMP-2B detects the mRNA of *Rvg*. **D**–**G** Quantitative assessment of *Gap43* (**D** and **F**) and *Snap25* (**E** and **G**) in HEK293T cells (**D** and **E**) and EVs (**F** and **G**) using RT-qPCR. **H** HA pulldown assays and representative Western blots of 5×FAD hippocampal tissues treated with EVs-TNGS. *n* = 3. **I**–**K** Representative Western blots (**I**) and their quantitative assessment (**J** and **K**) of protein levels of GAP43 (**I** and **J**) and SNAP25 (**I** and **K**) in treated 5×FAD mouse brains. EVs, extracellular vesicles; EVs-TN, extracellular vesicles targeting neural cells; EVs-TNGS, extracellular vesicles targeting neural cells overexpressing *Gap43* and *Snap25*; GAP43, growth-associated protein 43; mRNAs, messenger RNAs; SNAP25, synaptosome-associated protein 25; RT-qPCR, real-time quantitative reverse transcription-polymerase chain reaction; RVG, rabies virus glycoprotein

To investigate whether increases in *Gap43* and *Snap25* could alleviate the cognitive impairment of 5×FAD, we designed an EV-based system to overexpress *Gap43* and *Snap25* in the neural cells of the 5×FAD mouse brain (Fig. 5A). The EVs specifically targeting the neural population (EVs-TN) were engineered using HEK293T cells expressing LAMP-2B, an EV-derived membrane protein, fused with the neuron-specific RVG peptide. We first assessed whether the construction and transfection of the plasmids were successful. A pulldown assay using an anti-HA antibody demonstrated that HA-LAMP2B was highly expressed in transfected HEK293T cells (Fig. 5B). Additionally, Western blot results showed that HA-LAMP2B was present in HEK293T-derived EVs (Fig. 5B). *Rvg* expression in transfected HEK293T cells was measured using RT-qPCR analysis (Fig. 5C). These data confirm the successful construction of an engineered EV system expressing neuron-specific peptides in HEK293T cells.

### Overexpression of Gap43 and Snap25 in EV system targeting neural cells

**Fig. 6:**
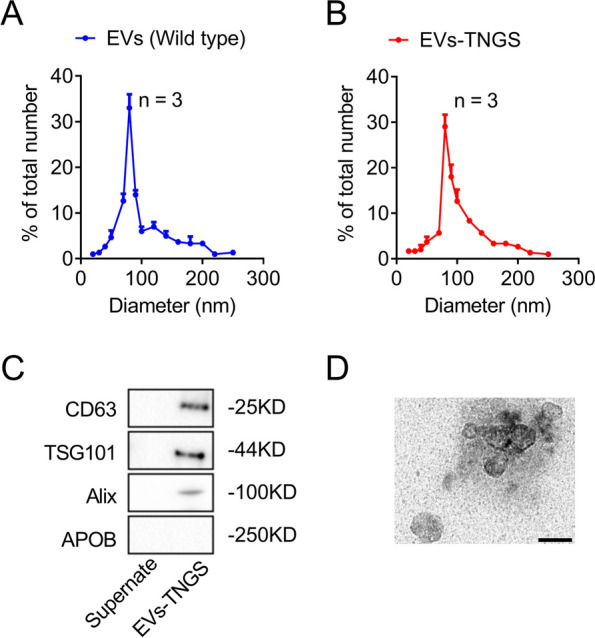
Characterization of EVs. **A**, **B** Size distribution of wild-type (**A**, 97.3 ± 42.8 nm) and transfected EVs (**B**, 99.5 ± 41.0 nm) measured using nanoparticle tracking analysis. **C** Protein levels of the EV markers Alix, TSG101, CD63, and APOB measured using Western blot analysis. **D** Transmission electron microscope image showing the distinctive spherical-shaped morphology of EVs. Scale bar = 100 nm. APOB, apolipoprotein B; EVs, extracellular vesicles; EVs-TNGS, extracellular vesicles targeting neural cells overexpressing *Gap43* and *Snap25*; TSG101, tumor susceptibility gene 101

Next, we overexpressed *Gap43* and *Snap25* in the engineered HEK293T cells. Our results showed that *Gap43* and *Snap25* mRNA levels were significantly higher in the total RNA extract from transfected HEK293T cells and EVs than in that of the control group (Fig. 5D–G), indicating that *Gap43* and *Snap25* were successfully overexpressed. These data suggested that *Gap43* and *Snap25* were successfully loaded into the EVs, and thereby, the EVs targeting neural cells overexpressing *Gap43* and *Snap25* were successfully constructed. To characterize the EVs, we confirmed that the number and size of EVs-TNGS were not altered compared to those of wild-type EVs (Fig. 6A, B). We also confirmed that Alix, TSG101, and CD63 were highly expressed in the EV-TNGS samples but not in the supernatants, while the negative marker, APOB, was absent in the EV-TNGS samples (Fig. 6C). In addition, the transmission electron microscope image revealed the distinctive spherical-shaped and nanosized morphology of EVs (Fig. 6D). Collectively, these findings indicated that the collection of EVs-TNGS was successful. To assess the long-term stability of EVs, we compared freshly harvested wild-type EVs and EVs-TNGS with those stored at – 80 °C for 30 days, examining their concentration, contaminant protein content, and size. Notably, no significant differences were observed between the groups of freshly harvested and 30-day-stored EVs (Additional file 1: Fig. S7), affirming their sustained stability over an extended storage period.

We then administered EVs-TNGS to 5×FAD mice via tail vein injection. Two weeks later, the mice were sacrificed, and the expression levels of GAP43 and SNAP25 in the hippocampal tissue were measured using an HA pulldown assay. GAP43 and SNAP25 were contained in the EV-TNGS group, while no signal was detected in the group treated with wild-type EVs (Fig. 5H), suggesting that EVs-TNGS were successfully internalized in the hippocampus. In addition, the hippocampal tissues of 5×FAD mice showed increased levels of GAP43 and SNAP25 in the EV-TNGS treatment group (Fig. 5I–K). These results indicated that EVs targeting neural cells successfully delivered *Gap43* and *Snap25* to the brains of 5×FAD mice. As EVs-TNGS contained *Rvg* mRNAs, we, therefore, performed RT-qPCR of *Rvg* mRNAs to assess the distribution of EVs-TNGS in the major organs of the sacrificed mice. The results showed that levels of EVs-TNGS were lowest in the heart, spleen, and muscle. The levels detected in the liver, lungs, and kidney were tenfold higher than those observed in the heart, spleen, and muscle, which was similar to previously published data [50]. Remarkably, the cortical and hippocampal levels surpassed 50% of those found in the liver, lungs, and kidney, which was higher than the levels reported for non-RVG-engineered EVs in prior studies [50]. These results underscore the distinct advantage of our EVs-TNGS method (Additional file 1: Fig. S8). Moreover, Caspase 3 activity measurement revealed a low apoptosis rate in HEK293T cells after transfection, as no differences were observed among the preparations for different EVs (Additional file 1: Fig. S9).

### EV-TNGS treatment improves memory performance in 5×FAD mice

**Fig. 7:**
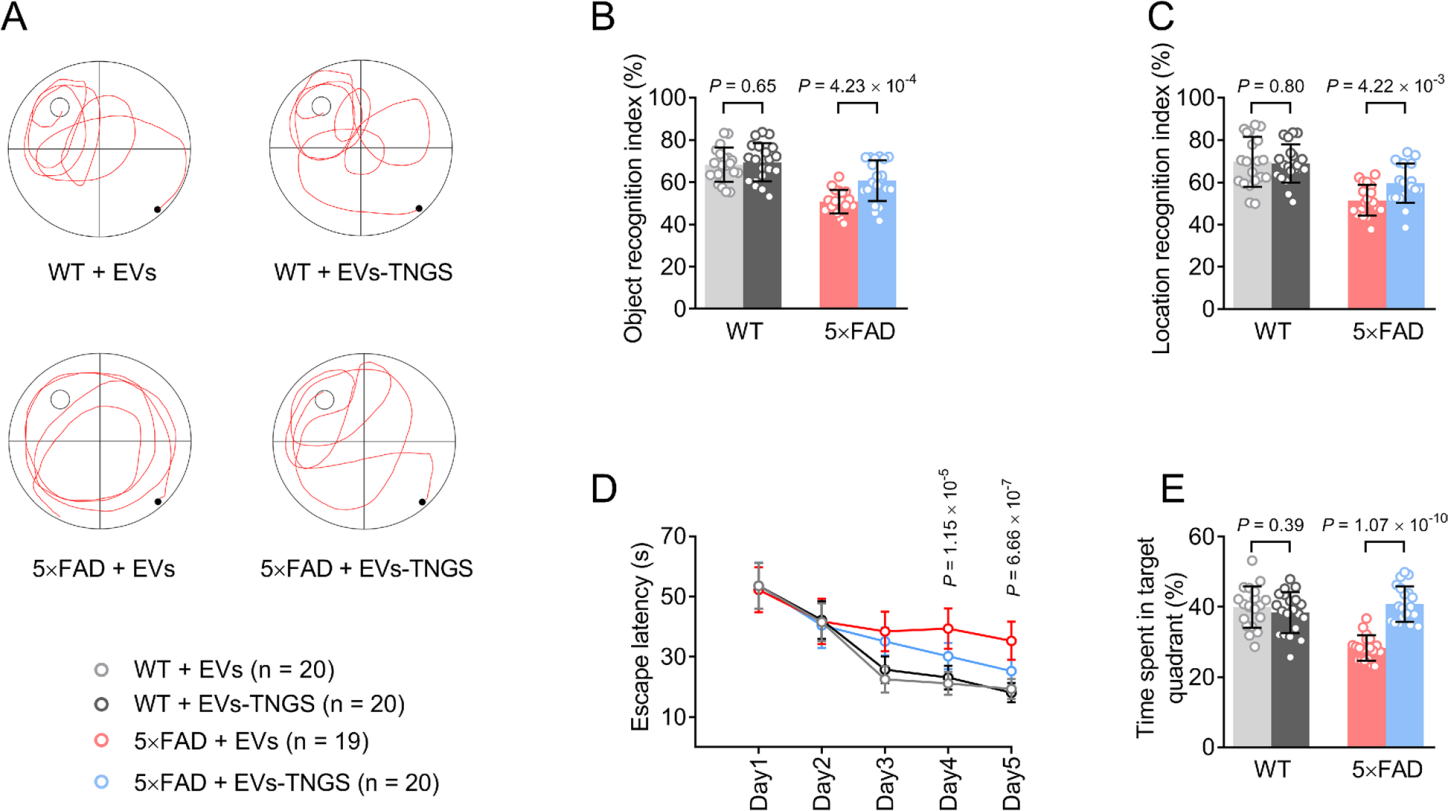
Improvement of memory performance in 5×FAD mice following EV treatment. **A** Representative trajectories of each group in the probe test of MWM. **B** NOR test was performed 1 month after treatment. **C** NOL test was performed 1 month after treatment. **D** The parameters of escape latency were recorded for 5 days to assess the cognitive function of mice. **E** The time spent in the correct quadrant was recorded in the probe test of MWM. * *P* < 0.05, 5×FAD+EVs-TNGS compared to 5×FAD+EVs. EVs, extracellular vesicles; EVs-TNGS, extracellular vesicles targeting neural cells overexpressing growth-associated protein 43 and synaptosome-associated protein 25; MWM, Morris water maze; NOR, novel object recognition; NOL, novel object location; WT, wild-type

The 5×FAD mice treated with EVs-TNGS showed a significantly higher recognition index in NOR and NOL tasks compared to those treated with wild-type EVs (all *P* < 0.05), while no significant differences were observed in the performance between wild-type mice treated with wild-type EVs and EVs-TNGS (Fig. 7B, C). We then investigated the spatial learning and memory abilities of mice using the MWM. EV-TNGS-treated 5×FAD mice had significantly shorter escape latencies on the 4^th^ and 5^th^ days of training compared to those treated with wild-type EVs (all *P* < 0.05), while no significant differences were observed in wild-type mice treated with wild-type EVs and EVs-TNGS (Fig. 7A, D). In the probe test, 5×FAD mice treated with EVs-TNGS spent more time in the target quadrant than mice treated with wild-type EVs (*P* < 0.05), indicating that treatment with EVs-TNGS can alleviate spatial learning disabilities in 5×FAD mice (Fig. 7A, E). Taken together, our findings suggest that EV-TNGS treatment can improve the recognition ability and spatial memory ability of 5×FAD mice.

### Treatment with EVs-TNGS increases the expression of GAP43 and SNAP25 and alleviates dendritic spine loss

**Fig. 8:**
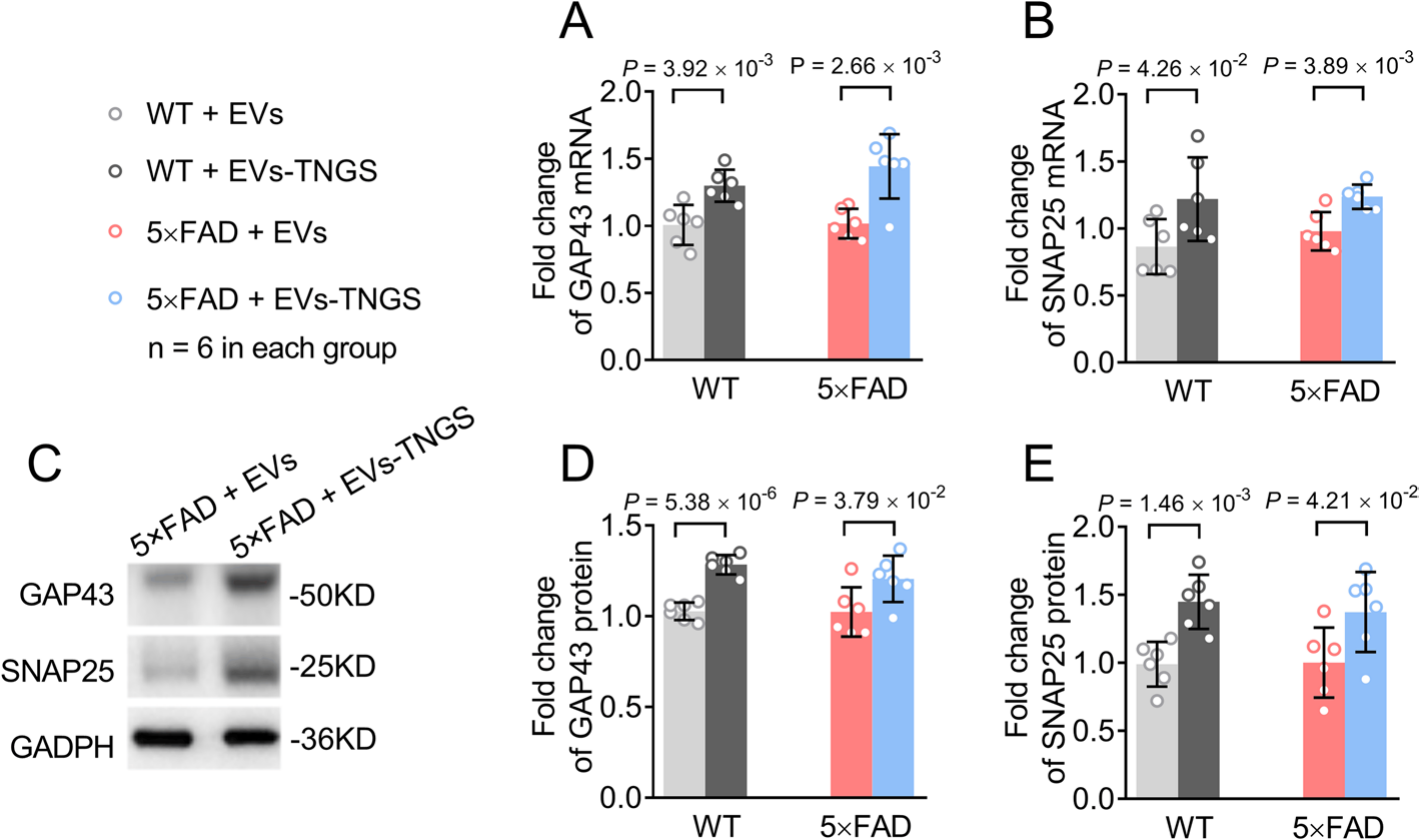
Increase in levels of GAP43 and SNAP25 in the 5×FAD mouse brains treated with EVs-TNGS. **A** Upregulation of *Gap43* mRNA was detected using RT-qPCR in EV-TNGS-treated hippocampal tissues. **B** Upregulation of *Snap25* mRNA was detected using RT-qPCR in EV-TNGS-treated hippocampal tissues. **C**–**E** Representative Western blots (**C**) and their quantitative assessment (**D** and **E**) show protein levels of GAP43 (**C** and **D**) and SNAP25 (**C** and **E**) in EV-TNGS-treated hippocampal tissues. EVs, extracellular vesicles; EVs-TNGS, extracellular vesicles targeting neural cells overexpressing *Gap43* and *Snap25*; GAP43, growth-associated protein 43; RT-qPCR, real-time quantitative reverse transcription-polymerase chain reaction; SNAP25, synaptosome-associated protein 25; WT, wild-type

**Fig. 9:**
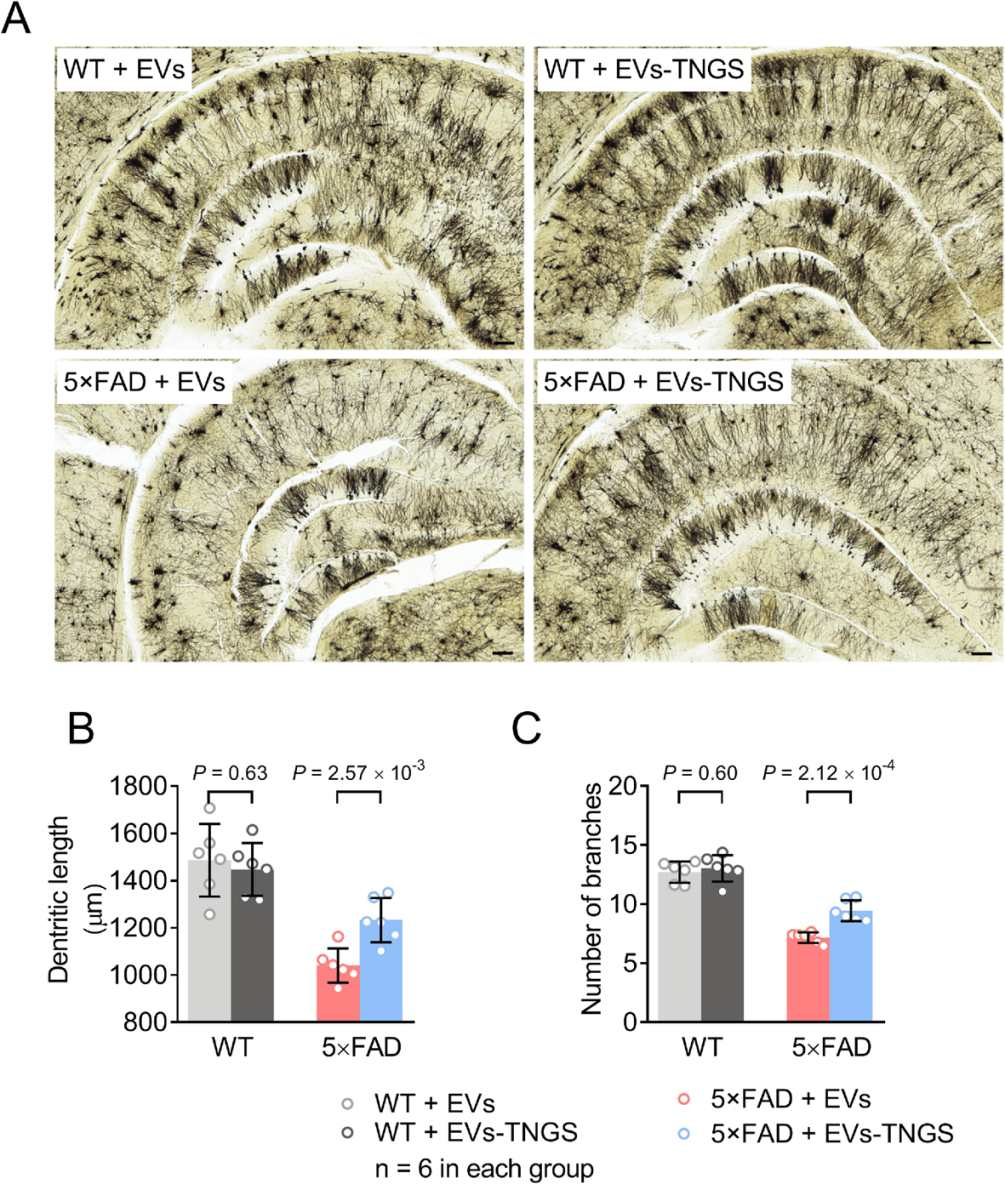
Increase in dendritic density in 5×FAD mouse brains treated with EVs-TNGS. Representative Golgi stains (**A**) and their quantitative data (**B** and **C**) show total dendritic length (**B**) and neuron branch number (**C**) in the hippocampus. Scale bar = 100 μm. EVs, extracellular vesicles; EVs-TNGS, extracellular vesicles targeting neural cells overexpressing growth-associated protein 43 and synaptosome-associated protein 25; WT, wild-type

To further confirm whether treatment with EVs-TNGS can increase the expression of GAP43 and SNAP25, we analyzed their expression levels using RT-qPCR and Western blot. Both mRNA and protein levels of GAP43 and SNAP25 were increased by the treatment, both in 5×FAD and wild-type mice (Fig. 8). To investigate whether the therapeutic effects are associated with improvements in the loss of the dendritic spine, Golgi staining and dendritic calculations were performed. Golgi-Cox staining showed that the dendritic length (Fig. 9A, B, Additional file 1: Fig. S10A), the number of branches (Fig. 9A, C, Additional file 1: Fig. S10A), and the spine density (Fig. 9A, Additional file 1: Fig. S10A, B) in 5×FAD mice were significantly reduced. However, these three parameters were improved by treatment with EVs-TNGS (Fig. 9, Additional file 1: Fig. S10). Taken together, these data suggest that treatment with EVs-TNGS may improve cognitive function in 5×FAD mice by elevating levels of GAP43 and SNAP25 and alleviating dendritic spine loss.

To evaluate whether the therapeutic effects depend on synaptic proteins per se or by simultaneously involving other AD pathologies, we measured the levels of Aβ42 and Aβ40 in the cortex and hippocampus of all mice (Additional file 1: Figs. S11 and S12). No significant differences were observed between the groups. The results indicate that *Gap43* and *Snap25* exert their effects independently of Aβ pathology.

## Discussion

In the present study, we demonstrated that GAP43, neurogranin, SNAP25, and synaptotagmin 1 were significantly changed in both EVs and CSF in patients with AD, and these changes corresponded to the severity of brain atrophy. In the 5×FAD mouse model, we confirmed that GAP43 and SNAP25 were decreased in the brains. Therefore, we constructed neural-targeting EVs overexpressing *Gap43* and *Snap25* mRNAs. The administration of EVs-TNGS successfully augmented the expression levels of GAP43 and SNAP25 in mouse brains and improved the cognitive performance of 5×FAD mice by increasing dendritic density. This study demonstrates the therapeutic potential of EV-based gene therapy for AD. To our knowledge, this is the first study to deliver synaptic protein mRNAs via EVs into the brains of AD mice with remarkable therapeutic effects. These findings provide new insights and pave the way for novel therapeutic approaches to AD.

The alteration of synaptic proteins observed in brain tissue, CSF, and neuronal-derived EVs in this study are consistent with our previous findings [22] and others [20, 21, 53–55]. Since neuronal-derived EVs originate from neurons, the reduction in synaptic proteins in brain tissue could result in the corresponding decrease in their levels within EVs [53]. Conversely, the elevated levels of synaptic proteins detected in CSF could be attributed to the continuous leakage of these proteins from brain tissue into the interstitial fluid that eventually drains into CSF [56]. In this context, the synchronized change of these proteins between brain tissue and neuronal-derived EVs highlights the potential of EVs as reliable carriers within CNS and the theoretical grounds for this study. Furthermore, the close relationship between the levels of synaptic proteins in EVs and hippocampal atrophy underscores their therapeutic role. This is further supported by the observed increase in dendritic density in 5×FAD mice following EVs-TNGS treatment.

Therapeutics based on mRNA have been investigated in detail in recent years and are considered a potential approach in precision medicine [57]. Naked mRNA delivery is limited by its high degradation rate and low cellular uptake capacity [58]. However, these limitations are circumvented by EV-mediated mRNA delivery [59]. EVs modified with RVG that specifically bind to the acetylcholine receptor [60] have been demonstrated to efficiently deliver miRNAs and mRNAs to the CNS [28, 47, 61]. With this promising approach, therapeutic mRNAs can be loaded and transported into the brain.

Synaptic dysfunction and loss are considered core features of AD [62]. We demonstrated that a group of synaptic proteins is altered in the CSF and neuronal-derived EVs in patients with AD [22, 63]. In line with these findings, synaptic proteins were significantly decreased in the brains of 5×FAD mice. Synapse loss is suggested to be an earlier and more initiative event in the pathophysiology of AD, rather than the consequence of neuronal loss, which represents a late hallmark of AD [7, 64]. Furthermore, a recent hypothesis posits a sequential progression involving Aβ deposition, subsequent synaptic dysfunction, tau aggregation, and eventual neurodegeneration [65], which is supported by a biomarker study showing that amyloid-related synaptic changes precede tau-related axonal degeneration [63]. According to this proposed model, synaptic damage induced by Aβ deposition triggers a response from glia to eliminate impaired synapses. As Aβ continues to accumulate, the severity of synaptic dysfunction intensifies, leading to tau hyperphosphorylation and the formation of tau tangles. This, in turn, results in axonal loss and eventual neurodegeneration [63, 65]. Nonetheless, tau accumulation also elicits synaptotoxicity at an early stage, as a close relationship between tau and synaptic dysfunction has been established through animal studies [66]. Furthermore, pathological proteins accumulate within synapses and propagate via synapses, potentially promoting the spread of pathology and degeneration throughout the brain [67]. Altogether, synaptic dysfunction results from intricate multi-directional interactions involving glial modifications as well as the toxic effects of Aβ and tau, supporting the notion that AD is principally a synaptopathy [63, 68]; however, the exact mechanism remains unclear. Moreover, previous studies indicate that synaptic dysfunction is a slow and protracted process that progresses from a reversible responsive phase to a compensatory phase and eventually to an irreversible failure phase, which is compatible with the evolution of AD [6, 69]. The findings of the current study show that the dendritic density and cognitive performance of 5×FAD mice treated with EVs-TNGS were improved, indicating that synaptic protein upregulation could restore synaptic function and thereby promote cognitive improvement in AD. Multiple studies [70–74] have collectively pointed to restoring synaptic proteins as a possible common pathway to improve cognitive performance. Moreover, a study showed that epigenetic editing of *Dlg4*, which encodes for post-synaptic density protein 95, rescued memory deficit in aged and AD mice [75]. In combination with these previous studies, our findings suggest that synaptic protein supplementation is a promising remedy for AD.

GAP43, also known as neuromodulin, is a presynaptic protein mainly expressed in the hippocampus and associative cortices in adults [76]. GAP43 is essential for axonal elongation and synaptogenesis and is a marker of neuronal growth and regeneration [17, 18]. Together with other synaptic components, GAP43 plays a crucial role in synaptic vesicle recycling, synaptic plasticity, and memory formation and storage [17]. Consistently, the increased expression of GAP43 in 5×FAD mice achieved with EVs-TNGS might enhance neuronal regeneration and synaptogenesis, thus leading to cognitive improvement. In addition, GAP43 appears to be a specific biomarker of AD, as it has been reported to be aberrantly expressed in AD [77, 78]. The positive correlation of GAP43 with Aβ and tau pathology further strengthens the AD-specific role of GAP43 [78]. Moreover, a study showed that the CSF level of GAP43 was significantly altered in apolipoprotein E (*APOE*) ε4 carriers, implying that early synaptic vulnerability may contribute to their susceptibility to AD [78]. Therefore, it might be feasible to develop preventive strategies based on synaptic protein supplementation in *APOE* ε4 carriers and other populations at risk for AD.

SNAP25 is an essential constituent of the soluble N-ethylmaleimide sensitive factor attachment protein receptor complex located in the presynaptic terminals, which is indispensable for synaptic transmission by initiating the exocytosis of synaptic vesicles and mediating neurotransmitter release [15]. Substantial evidence suggests a link between SNAP25 and learning and memory. Single-nucleotide polymorphisms of the *SNAP25* gene are associated with cognitive test performance in healthy individuals and subjects with AD and mild cognitive impairment [79, 80]. Animal studies have revealed that SNAP25 expression and activity in the hippocampal region are closely correlated with long-term potentiation and dendritic spine morphologies, which are considered the key elements of memory processing and cognitive function [81–83]. The noticeable cognitive enhancement observed in 5×FAD mice treated with EVs-TNGS might be partly attributed to improved synaptic plasticity and synapse reserve caused by SNAP25 upregulation. It is noteworthy that SNAP25 is also found to be altered in other neurological diseases, such as vascular dementia [84] and Parkinson’s disease [85, 86], indicating that synaptic protein supplementation strategy may be generalized beyond AD.

In the present study, two of the presynaptic proteins, SNAP25 and GAP43, were decreased in the brains of 5×FAD mice, while synaptotagmin 1 and the postsynaptic protein, neurogranin, did not show any significant changes, which is consistent with the finding that presynaptic alteration is preferentially involved in AD [87]. However, the levels of all four synaptic proteins in CSF and neuronal-derived EV were changed in patients with AD. This discrepancy might be explained by the differential expression of these synaptic proteins at different stages of AD. Specifically, neurogranin and synaptotagmin 1 might be in their compensatory phase, while SNAP25 and GAP43 might be in the responsive phase. This hypothesis is further supported by the non-significant differences observed in the levels of all four synaptic proteins when comparing individuals with an MTA score of 0 or 1. Theoretically, overexpression of neurogranin and synaptotagmin 1 can also contribute to improvement in synaptic density and function, as well as cognitive function, since they participate in memory processing and learning by regulating calcium dynamics, synaptic transmission, and synaptic plasticity [16, 19].

### Strengths and limitations

The major strength of this study lies in its derivation of ideas from our clinical findings. Based on clinical evidence demonstrating the downregulation of synaptic proteins in patients with AD, we proposed upregulating these proteins as a potential intervention strategy. Additionally, we developed an efficient vector capable of targeting the CNS for intervention purposes by leveraging the advantages of EVs. Finally, through the integration of clinical analyses and animal experiments, we validated the association between synaptic proteins and hippocampal atrophy, establishing a solid theoretical foundation for their therapeutic role. However, it is important to acknowledge certain limitations in our study. Firstly, our clinical study did not include individuals with mild cognitive impairment, which may result in a biased distribution of MTA scores. Additionally, MTA score evaluation is semiquantitative, and we did not conduct automatic volumetry analysis. Furthermore, we did not investigate other AD phenotypes such as cortical thinning and hypometabolism. Finally, the 5×FAD model used in our study, while demonstrating neuronal loss and synaptic dysfunctions absent in many other AD models, exhibits a rapid and aggressive AD pathology that does not fully represent the complexities of the human AD condition [88]. Therefore, the findings of our study should be interpreted cautiously. Unfortunately, the development of effective treatment strategies for AD is hindered by the lack of animal models that can replicate the full spectrum of characteristics observed in human AD. To enhance the translatability of research findings from preclinical models to human clinical trials, it is imperative to develop more clinically relevant AD models coupled with a deeper understanding of the phenotypes exhibited by these models.

## Conclusions

In conclusion, our study demonstrates that synaptic proteins in EVs are closely related to AD processes and synaptic protein upregulation can alleviate cognitive impairment in AD by promoting dendritic density. This study also highlights that EV-based mRNA therapy is a novel and promising direction for AD treatment, warranting further clinical trials in the future to confirm the results.

### Supplementary Information

**Additional file 1: Fig. S1.** Cutoff values of CSF biomarkers to determine AD. **Fig. S2.** RNA yield and purity of the EVs and HEK293T cells. **Fig. S3.** Effect of different dosages of EV-TNGS treatment on the mRNA levels of *Gap43* and *Snap25*. **Fig. S4.** Neuronal-derived EV levels of synaptic proteins in healthy controls. **Fig. S5.** CSF levels of synaptic proteins in healthy controls. **Fig. S6.** Reduction of GAP43 and SNAP25 levels in the hippocampus of 5×FAD mouse brain. **Fig. S7.** Stability of EVs after 30 days of storage. **Fig. S8.** Distribution of engineered EVs in the mice. **Fig. S9.** Levels of Caspase 3 in different preparations of EVs. **Fig. S10.** Increase in dendritic spine density in 5×FAD mouse brains treated with EVs-TNGS. **Fig. S11.** Levels of Aβ42 and Aβ40 in the mouse brains after EV treatment measured using Simoa assay. **Fig. S12.** Levels of Aβ42 and Aβ40 in the mouse brains after EV treatment measured using MSD assay. **Table S1.** Real-time quantitative reverse transcription-polymerase chain reaction (RT-qPCR) primers. **Table S2.** Correlation between MTA score with cognitive performance. **Supplementary Methods.** Protocol of real-time quantitative reverse transcription-polymerase chain reaction (RT-qPCR) analysis.

**Additional file 2:** **Date file.** Original blot images.

## Data Availability

The supporting data will be made available on reasonable request.

## Abbreviations

Aβ: Amyloid-β
AD: Alzheimer’s disease
ADAS-Cog: The Alzheimer’s Disease assessment scale cognitive subscale
ANOVA: Analysis of variance
APOB: Apolipoprotein B
APOE: Apolipoprotein E
CNS: Central nervous system
CSF: Cerebrospinal fluid
EVs: Extracellular vesicles
EVs-TN: Extracellular vesicles targeting neural cells
EVs-TNGS: Extracellular vesicles targeting neural cells overexpressing *Gap43* and *Snap25*
GAP43: Growth-associated protein 43
miRNAs: MicroRNAs
mRNAs: Messenger RNAs
MTA: Medial temporal lobe atrophy
MWM: Morris water maze
NOL: Novel object location
NOR: Novel object recognition
PBS: Phosphate-buffered saline
RVG: Rabies virus glycoprotein
SDS: Sodium dodecyl sulfate
Simoa: Single-molecule array
siRNAs: Short interfering RNAs
SNAP25: SYNAPTOSOME-associated protein 25
TSG101: Tumor susceptibility gene101
