# Supplementary material for: Delivering synaptic protein mRNAs via extracellular vesicles ameliorates cognitive impairment in a mouse model of Alzheimer’s disease
Source: BMC Med. 2024 Mar 25;22:138. doi: 10.1186/s12916-024-03359-2 (PMC10964680; doi:10.1186/s12916-024-03359-2)
Supplement: Supplementary file 1 — Additional file 1: Fig. S1. Cutoff values of CSF biomarkers to determine AD. Fig. S2. RNA yield and purity of the EVs and HEK293T cells. Fig. S3. Effect of different dosages of EV-TNGS treatment on the mRNA levels of Gap43 and Snap25. Fig. S4. Neuronal-derived EV levels of synaptic proteins in healthy controls. Fig. S5. CSF levels of synaptic proteins in healthy controls. Fig. S6. Reduction of GAP43 and SNAP25 levels in the hippocampus of 5×FAD mouse brain. Fig. S7. Stability of EVs after 30 days of storage. Fig. S8. Distribution of engineered EVs in the mice. Fig. S9. Levels of Caspase 3 in different preparations of EVs. Fig. S10. Increase in dendritic spine density in 5×FAD mouse brains treated with EVs-TNGS. Fig. S11. Levels of Aβ42 and Aβ40 in the mouse brains after EV treatment measured using Simoa assay. Fig. S12. Levels of Aβ42 and Aβ40 in the mouse brains after EV treatment measured using MSD assay. Table S1. Real-time quantitative reverse transcription-polymerase chain reaction (RT-qPCR) primers. Table S2. Correlation between MTA score with cognitive performance. Supplementary Methods. Protocol of real-time quantitative reverse transcription-polymerase chain reaction (RT-qPCR) analysis. [file 12916_2024_3359_MOESM1_ESM.docx]

Supplementary Information

**Title:**

Delivering synaptic protein mRNAs via extracellular vesicles ameliorates cognitive impairment in a mouse model of Alzheimer’s disease

Cai *et al.*

**

**

**Supplementary Figure 1.** **Cutoff values of CSF biomarkers to determine AD.** The cutoff values of CSF P-tau181/ Aβ42 (0.14) and Aβ42 (500 pg/mL) were used to determine AD. The dotted line (slope = 0.14) indicates the cutoff for P-tau181/Aβ42; 500 pg/ml in the horizontal axis indicates the cutoff value of Aβ42. Aβ, amyloid-β; AD, Alzheimer’s disease; CSF, cerebrospinal fluid; P-tau, phosphorylated tau.





**Supplementary Figure 2. RNA yield and purity of the EVs and HEK293T cells.** A RNA yield of EVs and HEK293T cells. B and C RNA purity was determined by optical density ratios 260/280 (B) and 260/230 (C). Both 260/280 and 260/230 of EVs and HEK293T cells are around 2, suggesting good RNA purity. EV: extracellular vesicle; OD: optical density





**Supplementary Figure 3.** **Effect of different dosages of EV-TNGS treatment on the mRNA levels of *Gap43* and *Snap25*.** A and B mRNA levels of *Gap43* (A) and *Snap25* (B) in the brains of 5×FAD mice were measured using RT-qPCR after EV-TNGS treatment with different dosages. Dosage of EVs-TNGS was presented as particles/ 0.2 mL of phosphate-buffered saline per mouse. EV-TNGS: extracellular vesicle targeting neural cells overexpressing *Gap43* and *Snap25*; GAP43: growth-associated protein 43; RT-qPCR: real-time quantitative reverse transcription-polymerase chain reaction; SNAP25: synaptosome-associated protein 25


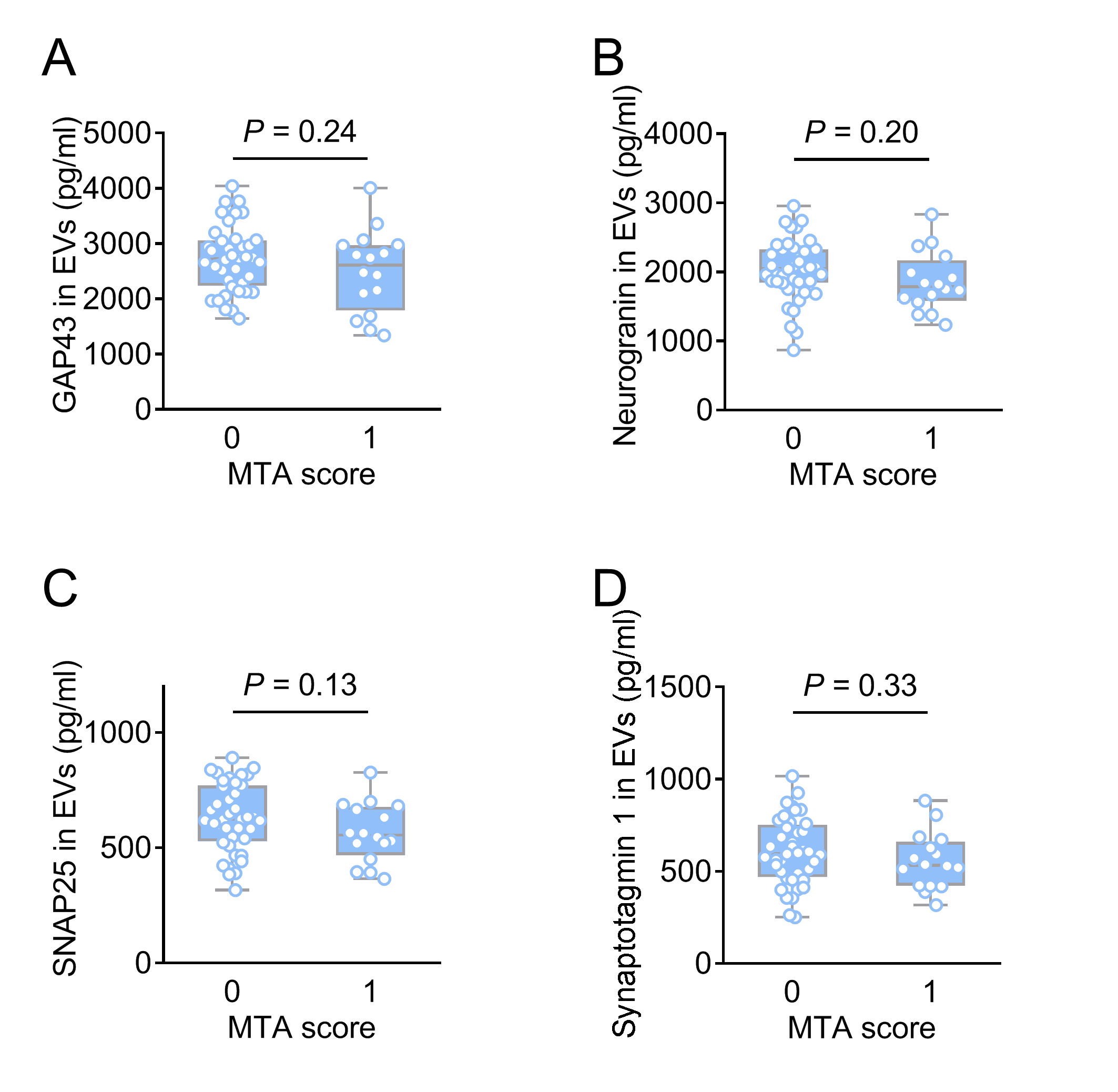


**Supplementary Figure 4. Neuronal-derived EV levels of synaptic proteins in** **healthy controls.** Neuronal-derived EV levels of GAP43 (A), neurogranin (B), SNAP25 (C), and synaptotagmin 1 (D) were measured in healthy controls with MTA scores of 0 and 1. EV: extracellular vesicle; GAP43: growth-associated protein 43; MTA: medial temporal lobe atrophy; SNAP25: synaptosome-associated protein 25





**Supplementary Figure 5. CSF levels of synaptic proteins in healthy controls.** CSF levels of GAP43 (A), neurogranin (B), SNAP25 (C), and synaptotagmin 1 (D) were measured in healthy controls with MTA scores of 0 and 1. CSF: cerebrospinal fluid; GAP43: growth-associated protein 43; MTA: medial temporal lobe atrophy; ns: no significance; SNAP25: synaptosome-associated protein 25


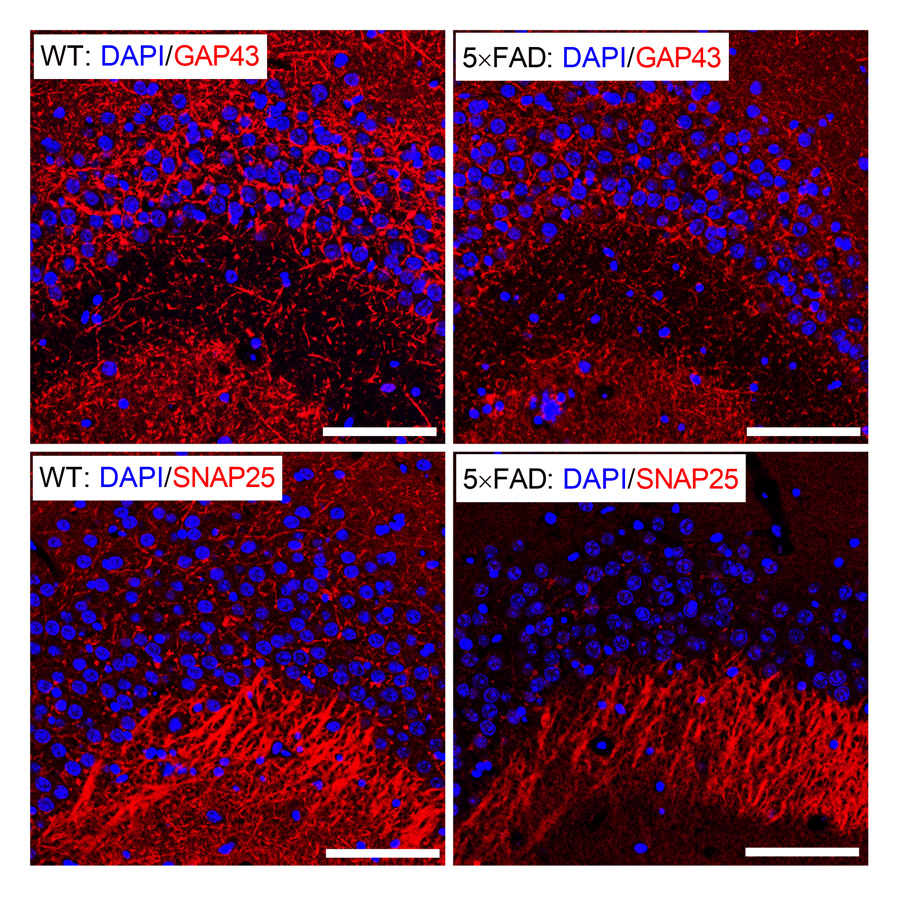


**Supplementary Figure 6. Reduction of GAP43 and SNAP25 levels in the hippocampus of 5×FAD mouse brain.** Representative images of immunofluorescent staining show reduced protein levels of GAP43 and SNAP25 in the hippocampus CA3 area of 5×FAD mice. GAP43: growth-associated protein 43; SNAP25: synaptosome-associated protein 25


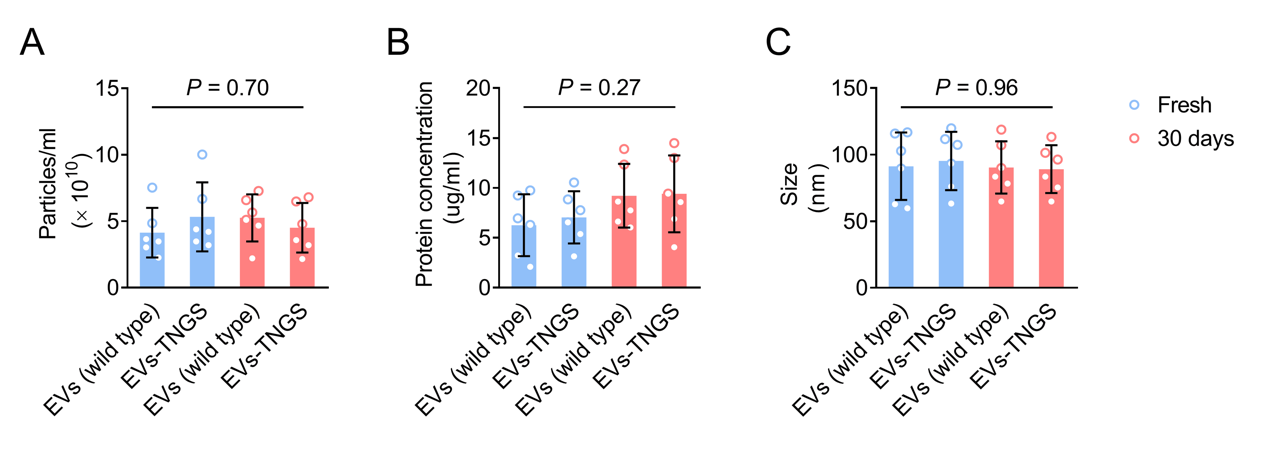


**Supplementary Figure 7. Stability of EVs after 30 days of storage.** Comparison between freshly harvested wild-type EVs and EVs-TNGS with those stored for 30 days in the concentration (A), protein content (B), and size (C). EVs: extracellular vesicles; EVs-TNGS: extracellular vesicles targeting neural cells overexpressing growth-associated protein 43 and synaptosome-associated protein 25





**Supplementary Figure 8. Distribution of engineered EVs in the mice.** Quantitative assessment of *Rvg* to assess the distribution of engineered EVs in the brain and other major organs in the mice. RVG, rabies virus glycoprotein





**Supplementary Figure 9.** **Levels of Caspase 3 in different preparations of EVs.** Quantitative assessment of Caspase 3 in preparations for different EVs after transfection at EV collection. X-axis indicates different preparations for different types of EVs. TN, targeting neural cells; TNGS, targeting neural cells overexpressing growth-associated protein 43 and synaptosome-associated protein 25; WT: wild-type





**Supplementary Figure 10. Increase in dendritic spine density in 5×FAD mouse brains treated with EVs-TNGS.** A and B Representative Golgi stains (A) and their quantitative data (B) of spine density in the hippocampus. Scale bar = 10 μm. EVs: extracellular vesicles; EVs-TNGS: extracellular vesicles targeting neural cells overexpressing growth-associated protein 43 and synaptosome-associated protein 25; WT: wild-type

**

**

**Supplementary Figure 11.** **Levels o****f** **Aβ42 and Aβ40 in the mouse brains** **after EV treatment measured using Simoa assay.** A-D Quantitative assessment of Aβ42 (A and B) and Aβ40 (C and D) in the cortex (A and C) and hippocampus (B and D) in the brains of mice of WT and 5×FAD treated with wild-type EVs and EVs-TNGS, which is measured using Quanterix Simoa assay. Aβ, amyloid-β; EVs: extracellular vesicles; EVs-TNGS, extracellular vesicles targeting neural cells overexpressing growth-associated protein 43 and synaptosome-associated protein 25; WT: wild-type





**Supplementary Figure 12. Levels of Aβ42 and Aβ40 in the mouse brains after EV treatment measured using MSD assay.** A-D Quantitative assessment of Aβ42 (A and B) and Aβ40 (C and D) in the cortex (A and C) and hippocampus (B and D) in the brains of mice of WT and 5×FAD treated with wild-type EVs and EVs-TNGS, which is measured with MSD assay using 6E10. Aβ, amyloid-β; EVs: extracellular vesicles; EVs-TNGS, extracellular vesicles targeting neural cells overexpressing growth-associated protein 43 and synaptosome-associated protein 25; WT: wild-type

**Supplementary Table 1. Real-time quantitative reverse transcription-polymerase chain reaction (RT-qPCR) primers.**

| Gene | Forward Primer | Reverse Primer |
| --- | --- | --- |
| *Gapdh* | GAAGGGCATCTTGGGCTACAC | GTTGTCATTGAGAGCAATGCCA |
| *Gap43* | CTGCTACTACCGATGCAGCC | CAGCACTTTCTGTCTCCGCT |
| *Snap25* | AATGATGCCCGGGAAAATGAG | GATCTGGCGATTCTGGGTGT |
| *Rvg* | TCGATACACCATTTGGATGCCCGAGAATCCGAGACCAGGGACACCTTGTGACATTTTTACCAATAGCAGAGGGAAGAGAGCATCCAACGGGT | CCGGACCCGTTGGATGCTCTCTTCCCTCTGCTATTGGTAAAAATGTCACAAGGTGTCCCTGGTCTCGGATTCTCGGGCATCCAAATGGTGTA |

**Supplementary Table 2. Correlation between MTA score with cognitive performance.**

| **Cognitive tests** | **r** | ***P*** |
| --- | --- | --- |
| MMSE | -0.93 | 1.69 x 10^-48^ |
| ADAS-Cog | 0.89 | 6.69 x 10^-29^ |

Abbreviations: ADAS-Cog, the Alzheimer's Disease assessment scale cognitive subscale; MMSE, Mini-Mental State Examination; MTA, medial temporal lobe atrophy

**Supplementary Methods**

**Protocol of** **real-time quantitative reverse transcription-polymerase chain reaction (RT-qPCR) analysis**

1. The ultraviolet spectrophotometer was used to verify the RNA isolation.
2. The components in the reverse transcription system:

Reverse transcription component list

| Component | Volume/reaction |
| --- | --- |
| 4X gDNA wiper Mix | 4 μl |
| Oligo(dT)_23_VN (10 µM) | 1 μl |
| Random hexamers (50 ng/µl) | 1 μl |
| DEPC-treated water | to 20 μl |
| Template RNA | 1 μg |
| 5X HiScript Ⅱ Select qRT SuperMix Ⅱ | 4 μl |
| Total volume | 20 μl |

1. The solution was gently vortexed and then incubated at 50°C for 15 min, 85°C for 5 sec, and kept at 4°C until use.
2. The components of the qRT-PCR system:

Ingredient list

| Component | Volume/reaction |
| --- | --- |
| 2X AceQ Universal SYBR qPCR Master Mix | 10 μl |
| 10 μM mRNA-specific forward primer | 0.4 μl |
| 10 μM mRNA-specific reverse primer | 0.4 μl |
| Diluted cDNA* | 2 μl |
| DEPC-treated water | 7.2 μl |
| Total volume | 20 μl |

*cDNA dilution ratio is 1:7

1. The qRT-PCR procedures:

Cycling Program

| Step | Time | Temperature |
| --- | --- | --- |
| Initial activation step | 5 min | 95ºC |
| Cycling: |  |  |
|  | 10 s | 95ºC |
|  | 30 s | 60ºC |
| Cycle number | 40 cycles |  |
| Melting curve |  |  |
|  | 15 s | 95ºC |
|  | 60 s | 60ºC |
|  | 15 s | 95ºC |

1. The calculation of fold change of mRNAs:

ΔCT = CT(target mRNA) − CT(Cel-GAPDH)

ΔΔCT = ΔCT(target sample) − ΔCT(control sample)

Fold change of target mRNA =2^-ΔΔCt^
