# Supplementary material for: Delivering synaptic protein mRNAs via extracellular vesicles ameliorates cognitive impairment in a mouse model of Alzheimer’s disease
Source: BMC Med. 2024 Mar 25;22:138. doi: 10.1186/s12916-024-03359-2 (PMC10964680; doi:10.1186/s12916-024-03359-2)

Representative western blotting image of GAP43 in the hippocampus of 5xFAD mouse

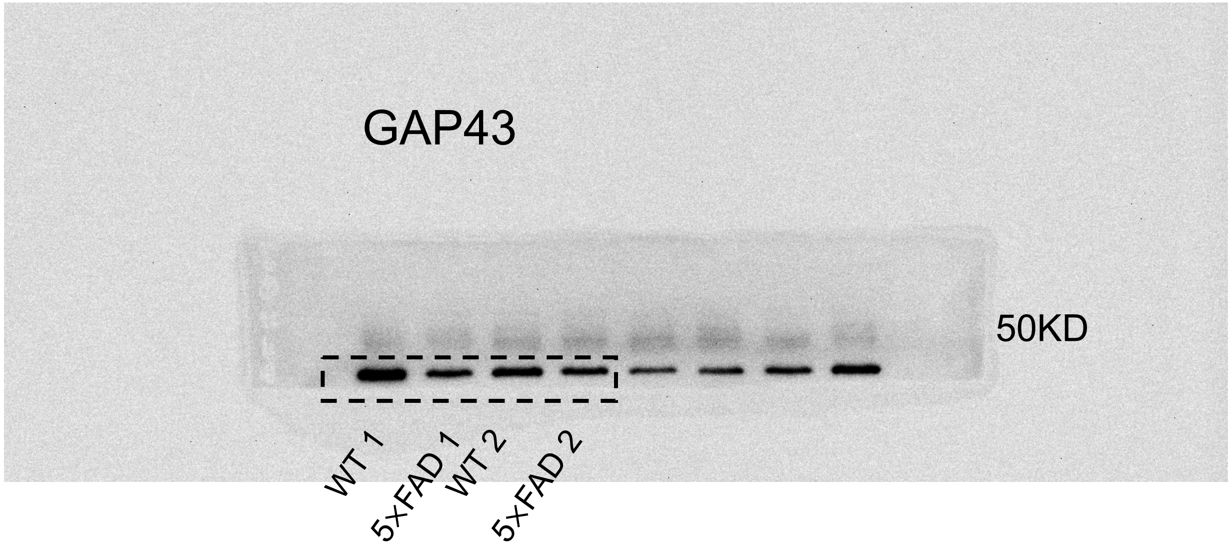

Representative western blotting image of SNAP25 in the hippocampus of 5xFAD mouse

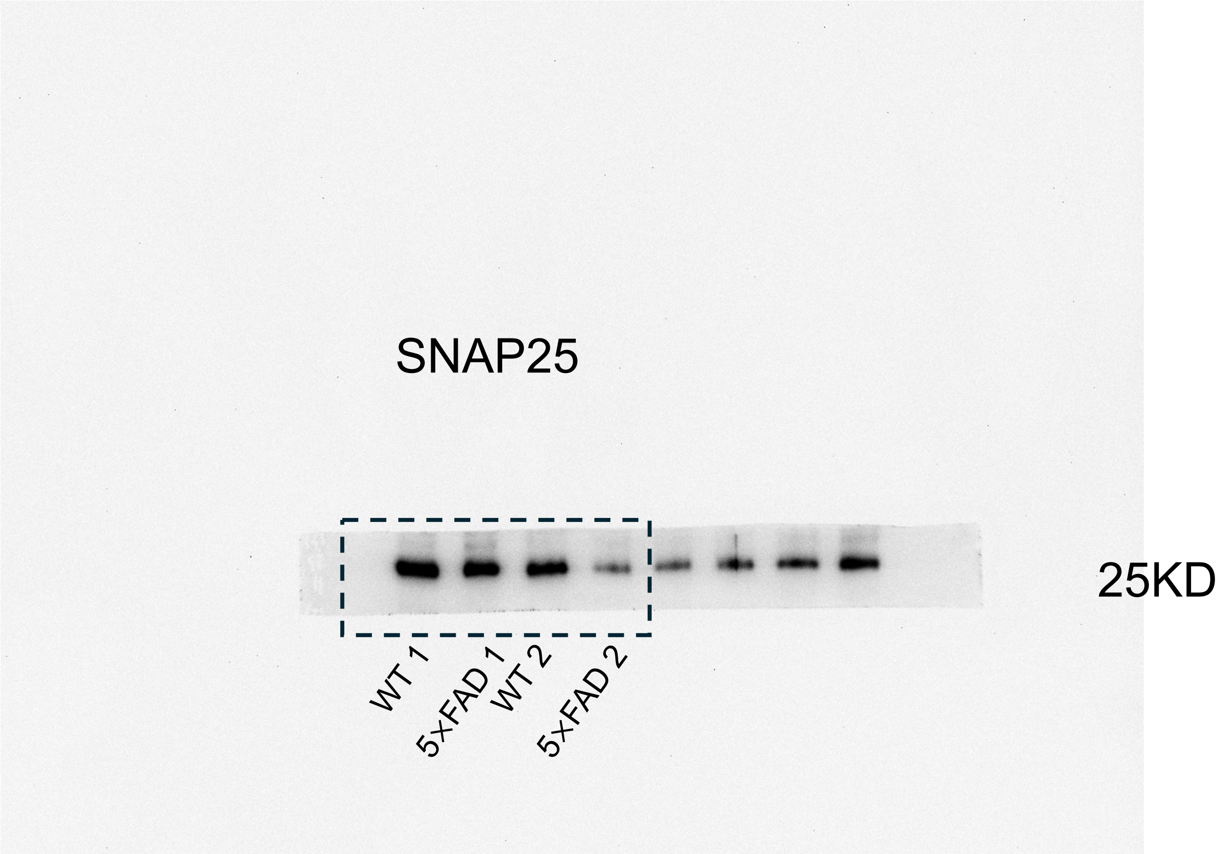

Representative western blotting image of Synaptotagmin 1 in the hippocampus of 5xFAD mouse

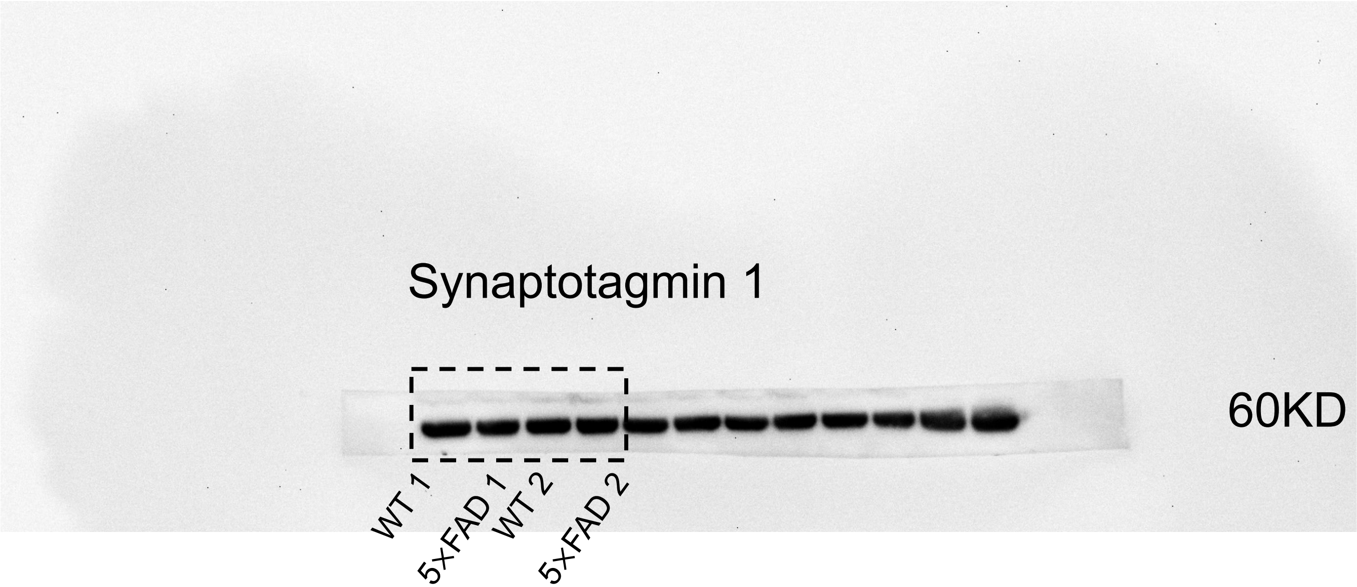

Representative western blotting image of Neurogranin in the hippocampus of 5xFAD mouse

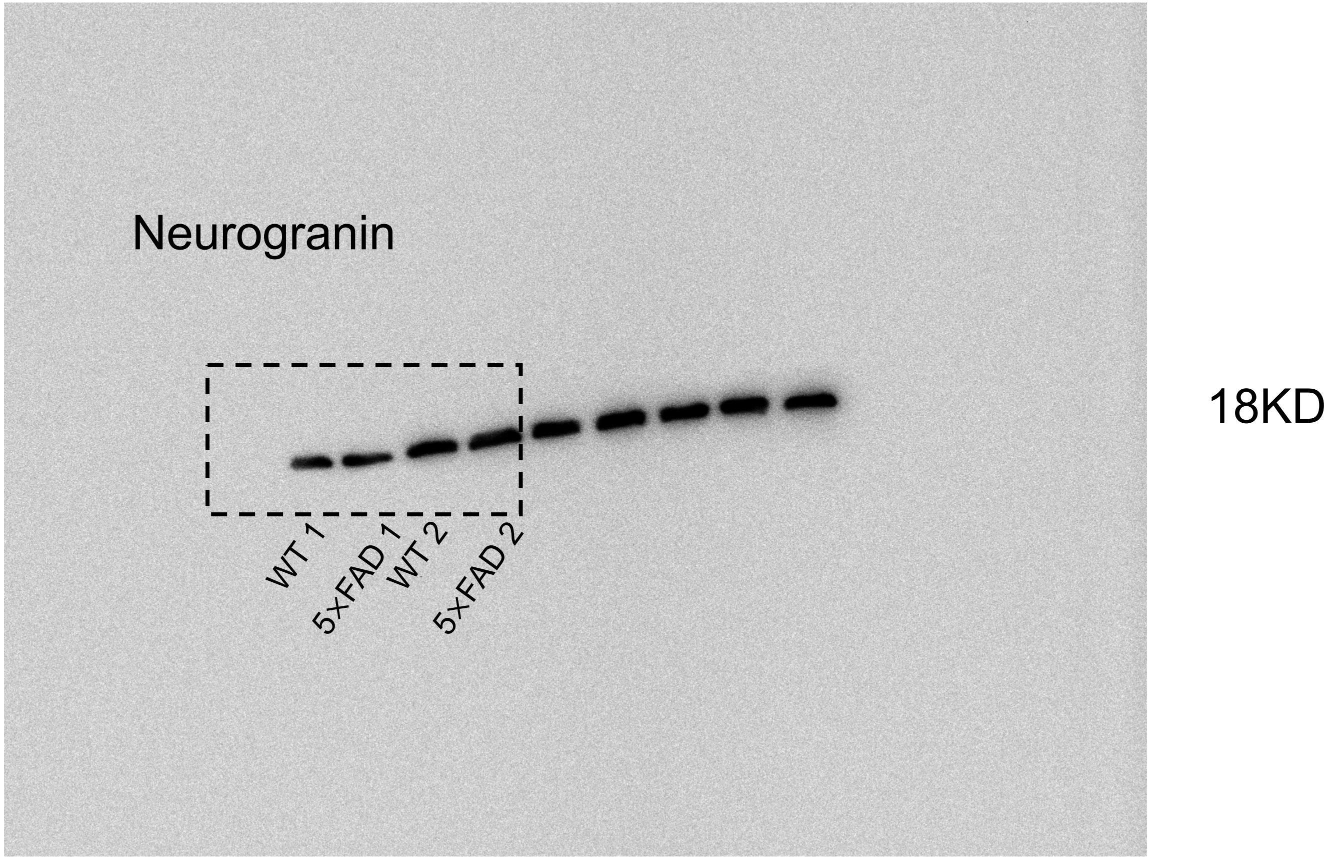

Representative western blotting image of GAPDH in the hippocampus of 5xFAD mouse

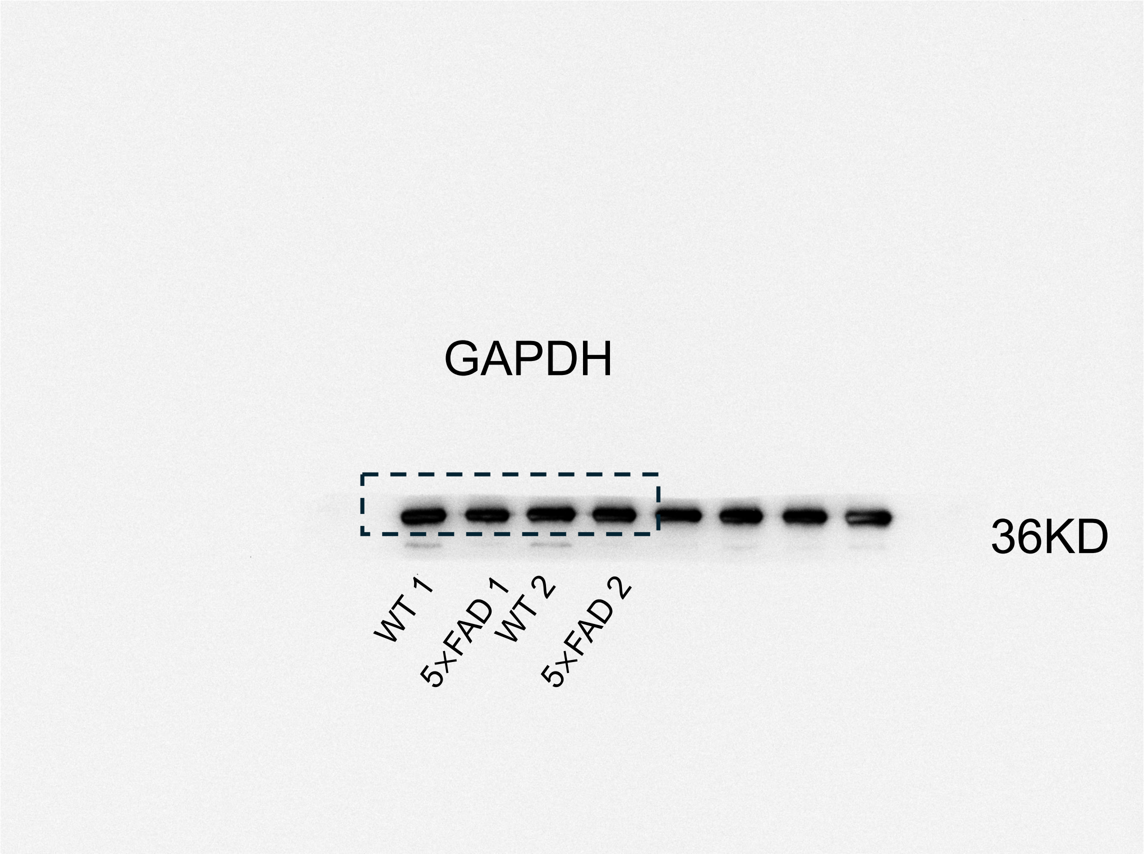

Representative western blotting images of protein levels of GAP43 and SNAP25 in treated 5xFAD mouse brains

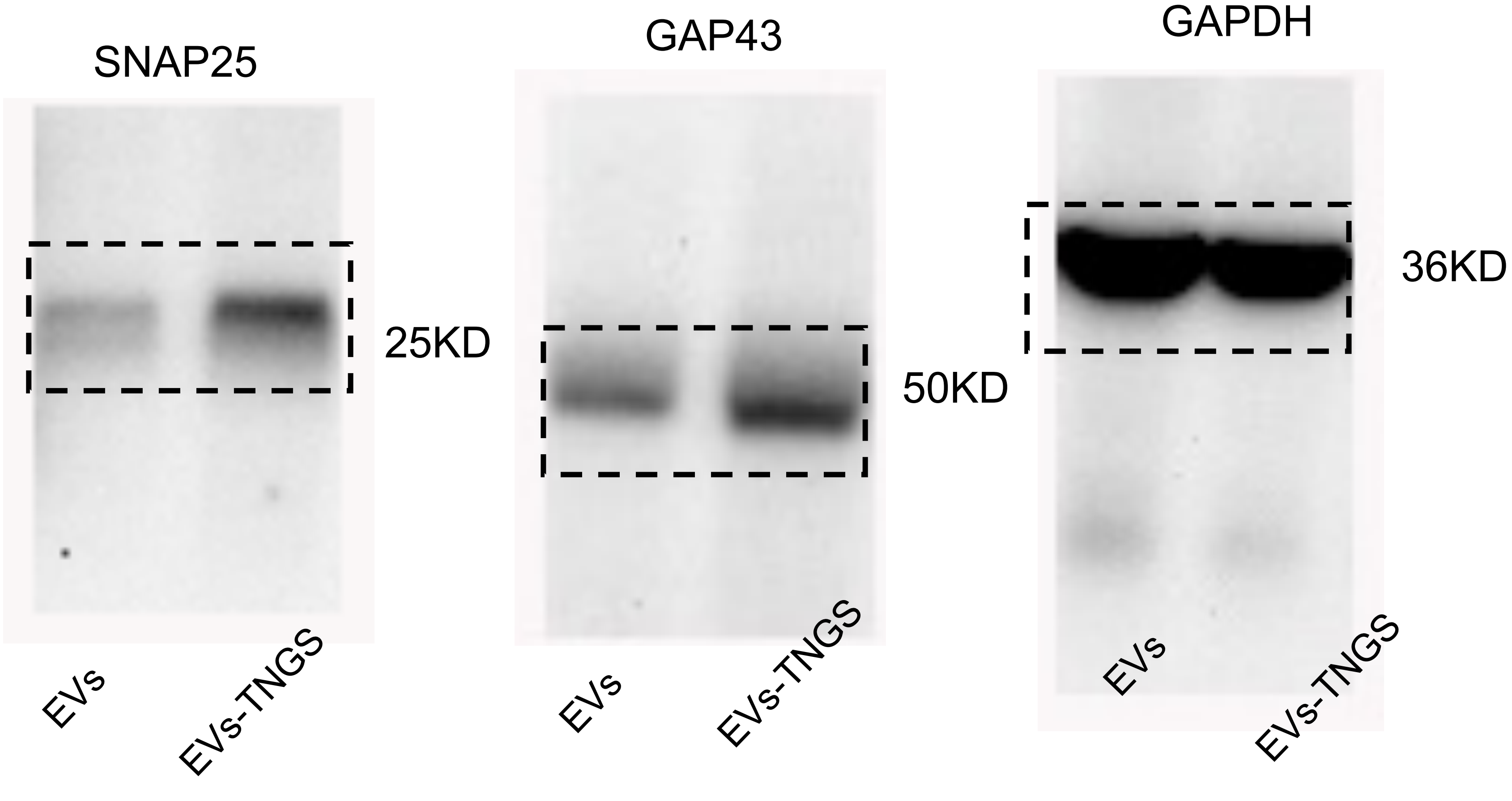

Representative western blotting image of protein levels of GAP43 and SNAP25 in EV-TNGS-treated hippocampal tissues in 5xFAD mice.

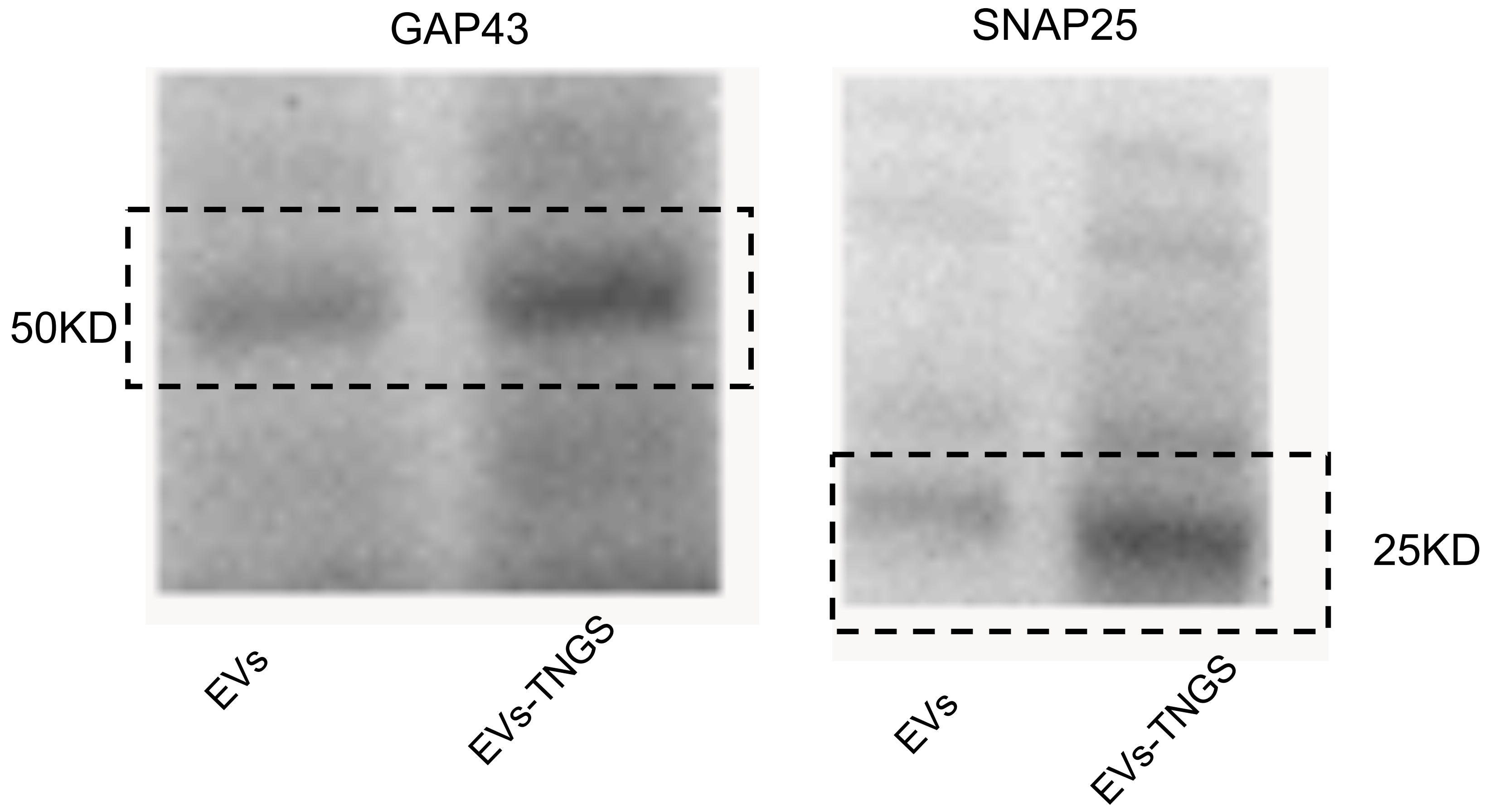

Representative western blotting image of protein levels of TSG101, CD63 and Alix in EV-TNGS.

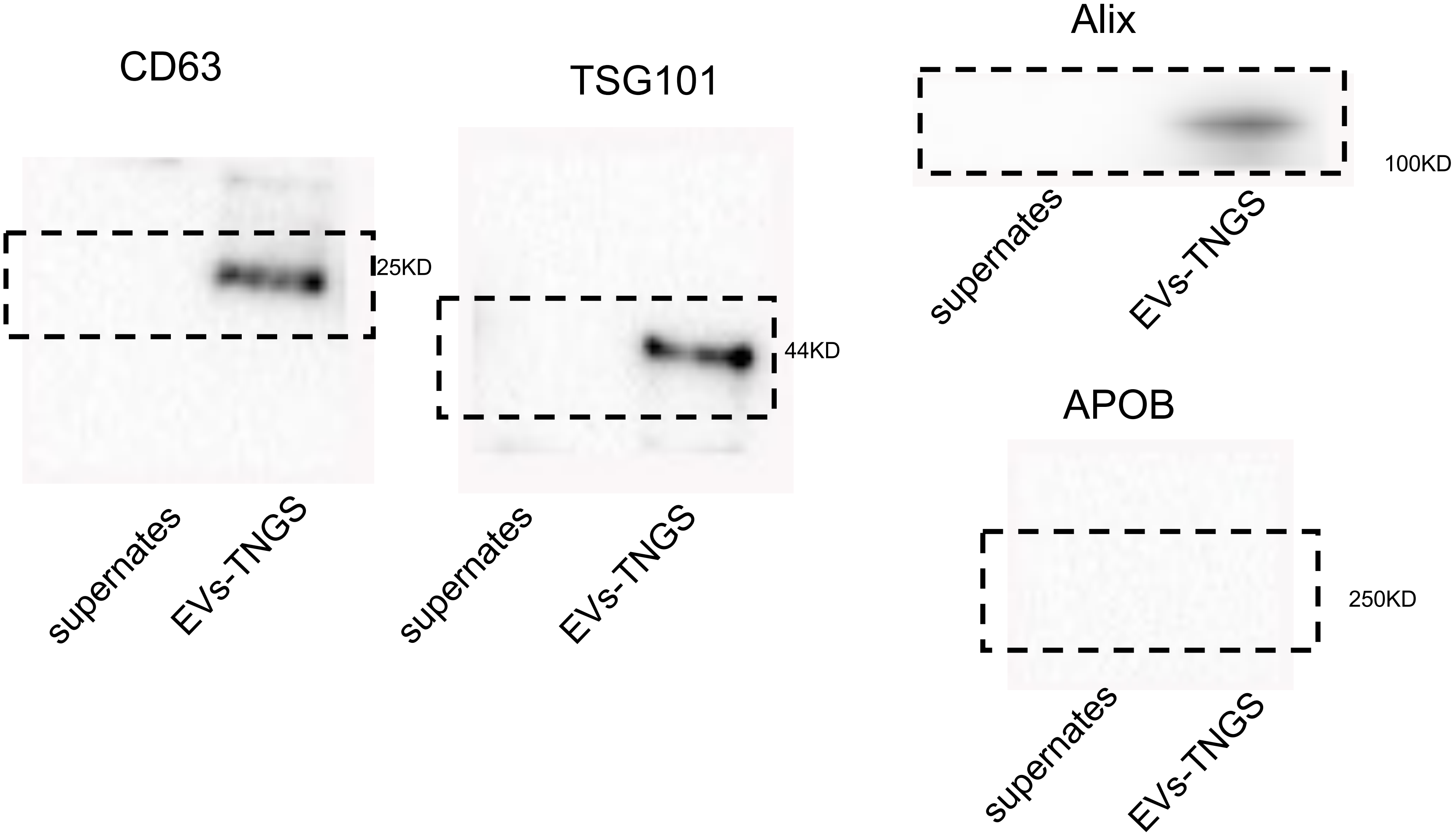

Supplement: Supplementary file 2 — Additional file 2: Date file. Original blot images. [file 12916_2024_3359_MOESM2_ESM.pdf]
